# How stress-related factors affect mental wellbeing of university students *A cross-sectional study to explore the associations between stressors*, *perceived stress*, *and mental wellbeing*

**DOI:** 10.1371/journal.pone.0275925

**Published:** 2022-11-07

**Authors:** Sybren Slimmen, Olaf Timmermans, Kalina Mikolajczak-Degrauwe, Anke Oenema

**Affiliations:** 1 Department of Health Promotion, Care and Public Health Research Institute (CAPHRI), Maastricht University, Peter Debyeplein, HA Maastricht, The Netherlands; 2 Research Group Healthy Region, HZ University of Applied Sciences, Edisonweg, NW Vlissingen, The Netherlands; 3 Centre for Research and Innovation in Health, Antwerp University, Universiteitsplein, Wilrijk, Belgium; Polytechnic Institute of Coimbra: Instituto Politecnico de Coimbra, PORTUGAL

## Abstract

**Background:**

Lowered mental wellbeing of students is a growing health and societal problem. Experiencing high levels of stress for a longer period of time has been associated with an increased risk for lower mental wellbeing and mental health problems. To reduce stress and improve mental wellbeing it is important to understand how various sources of stress are related with mental wellbeing and which factors can buffer the impact of stress on mental wellbeing.

**Objectives:**

Deriving from a conceptual model the aim of the study was to explore 1) the association of underlying stressors (academic pressure, family circumstances, side-activity pressure, and financial situation) with perceived stress and mental wellbeing, 2) whether perceived stress mediates the association between the sources of stress and mental wellbeing and 3) whether loneliness, self-esteem, personality and coping styles buffer or reinforce the impact of perceived stress on mental wellbeing.

**Method:**

A cross-sectional survey design was used among students of an University of Applied Sciences and conducted between November 16, 2020, and January 18, 2021. Study variables were mental wellbeing, perceived stress, academic pressure, financial pressure, family pressure and side-activity pressure, coping style, self-esteem, loneliness, personality. The questionnaire was constructed using validated measures. Simple and multiple linear regression analyses were conducted to assess the association between perceived stress, sources of stress and mental wellbeing. Mediation and moderation processes were explored using Hayes PROCESS models.

**Results:**

A total of 875 university students (37,2% male, 62,3% female, mean age 21,6) participated. Perceived stress had a strong negative association with mental wellbeing (unstandardized regression coefficient (*b)* = -.848, *p* < .001; *r =* -.667, *p* < .01), explaining 45% of the variance. Academic pressure (*b* = -8.014, *p* < .01), family pressure (*b* = -3.189, *p* < .01), side-activity pressure (*b* = -3.032, *p* < .01) and financial pressure (*b* = -2.041, *p* < .01) all had a negative impact on mental wellbeing. This effect was mediated by perceived stress, but a direct effect remained for academic pressure (*b* = -3.306, *p* < .01) and family pressure (*b* = -1.130, *p* < .01). Significant interaction effects between perceived stress and mental wellbeing were found for approach coping (low = -.93, *p* < .01; high = -.64, *p* < .01) and emotional stability (low = -.81, *p* < .01; high = -.64, *p* < .01).

**Conclusion:**

Perceived stress has a major impact on students’ mental wellbeing. Underlying stressors were mediated by perceived stress, but direct effects were also found. To protect the mental wellbeing of students, it is urgent to reduce perceived stress, suppress underlying stressors and make students more resilient through the development of found buffers, such as approach coping.

## Introduction

The mental wellbeing of students is under pressure, and this is a serious health and societal problem. Recent research has shown that students are a ’very high-risk population’ for mental health problems and disorders [1,2]. Mental wellbeing is more than just the absence of mental illness, but also includes psychological functioning, life satisfaction, and ability to develop and maintain mutually beneficial relationships [3]. Increased stress is considered an important cause for lowered mental wellbeing, as persistent stress is associated with an increased risk of mental disorders, a deteriorated quality of life and a decrease in study success [4–7]. In the Netherlands, recent studies have shown that over 60% of the students report excessive levels of stress [8–10]. In addition, more than half suffer from burnout-related complaints, such as emotional exhaustion and avoidance of social contacts [11]. To alleviate the problem and improve mental wellbeing of students it is important to increase our understanding of how perceived stress and underlying sources of stress are related to mental wellbeing and what factors can buffer the negative effects of stress on mental wellbeing. These insights can lead to the identification of target points for preventive interventions [10,12].

Stress is described as the way one deals with expectations and pressure from the environment that can be experienced as threatening or overwhelming by an individual [13]. Perceived stress is the extent to which certain situations are judged to be stressful [14]. The moment one falls short in personal resources to alleviate the perceived stress (e.g. lack of coping) there is a risk for a decreased mental wellbeing [15]. Perceived stress has a strong influence on the level of students’ mental wellbeing [16]. Evidence is available for an increased perceived stress level of university students in the past years [17,18]. Research has mainly been conducted among medical education (e.g., medicine, nursing, dentistry and psychology), but similar findings have been made for other educational programs [19]. In recent studies, several factors have been identified as underlying stressors having both a direct- and indirect effect on mental wellbeing. Academic- and performance pressures were reported in various educational profiles and seem to be the most influential determinants of perceived stress [10,20,21]. In addition, perceived stress has been found to strongly correlate with female gender [22], family circumstances [23], lack of leisure or side-activities [24], financial situation [25], self-esteem [26], coping style and study satisfaction [27]. Perceived stress has also been found to be a significant mediator in the association between emotional intelligence and indicators of mental wellbeing [28]. Similar mediation effects were found in the relation between social skills and mental wellbeing [29].

The impact of perceived stress on mental wellbeing, however, is complex and depends on internal and external personal resources, such as coping and social support [1]. The degree to which stress is perceived depends on underlying factors or stressors. Loneliness can lead to deteriorating personal resources, increasing the association of perceived stress and mental wellbeing. The impact of loneliness on mental wellbeing was especially found among international students, due to a lack of support coping and social contacts [30]. Personality also affects the association between perceived stress and mental wellbeing. A significant positive association was found between self-critical perfectionism, depression and stress symptoms [31], whereas students who are open to new experiences and environments, were found to experience significantly less stress and were more able to adapt to new environments [32]. Finally, applying the right coping style is crucial in dealing with stress, by preventing the development of its (potential) deleterious consequences [21]. The number of mental health problems experienced by students is related to their ability to cope with stressors [33,34]. Problem-focused coping, also named approach coping, seems to moderate the relation between perceived stress and mental wellbeing [35]. At low levels of problem-focused coping, perceived stress had a greater impact on mental wellbeing than at high levels of problem-focused coping.

**Fig 1:**
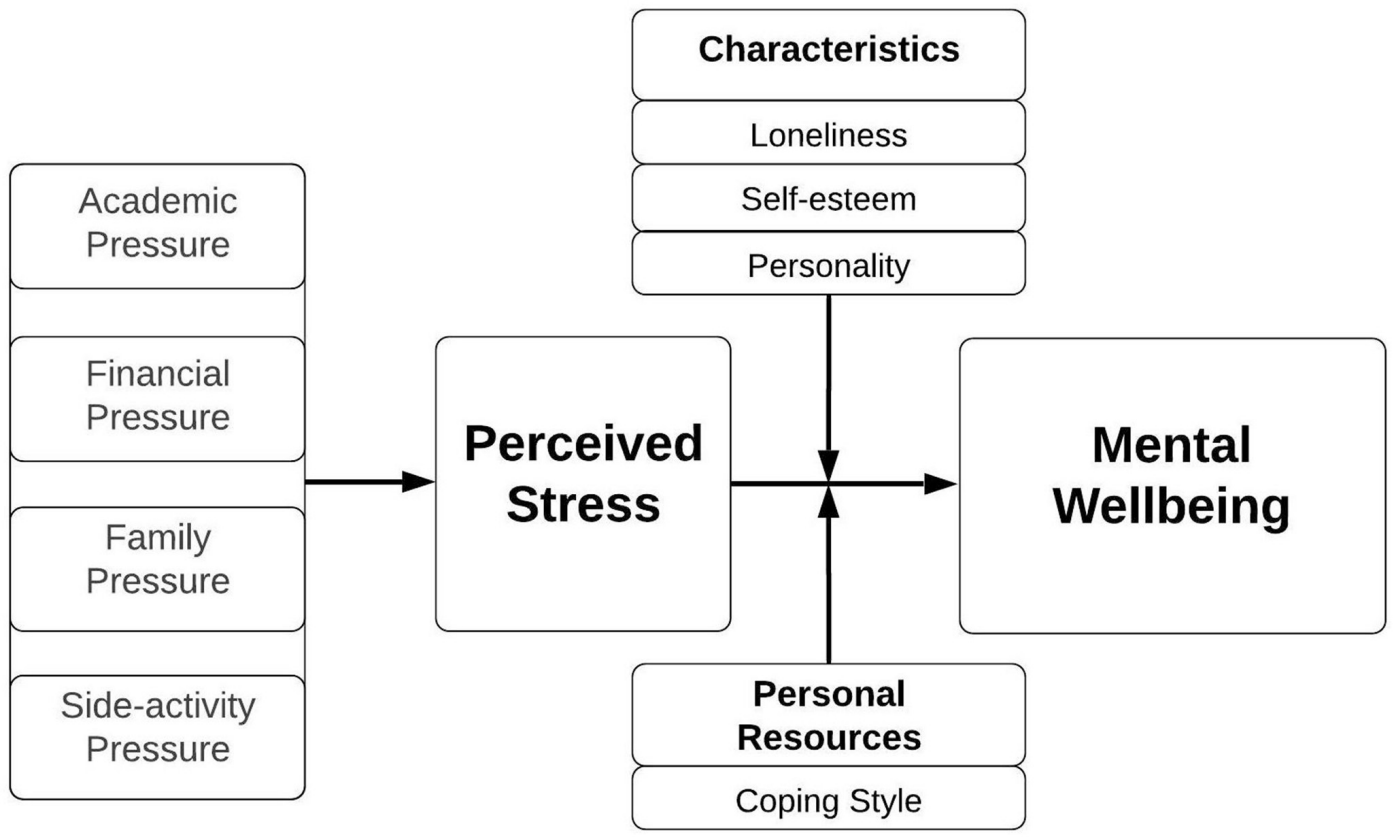
Conceptual model of the relationship between underlying stressors, perceived stress, characteristics, personal resources, and mental wellbeing.

Even though there is already evidence on the association between stressors, perceived stress, mental wellbeing and potential stress-buffering factors, there is a lack of insight in how all these factors link together. Based on the evidence of underlying factors, a conceptual model has been developed (Fig 1). The conceptual model presents the expected relations between underlying stressors, perceived stress, personal characteristics, personal resources and mental wellbeing. The structure of the conceptual model is partly derived from the Job Demands-Resources model designed by Demerouti, Bakker, Nachreiner, and Schaufeli and the Student Wellbeing model designed by Gubbels and Kappe [36,37]. These models reflect the balance between stressors and personal resources to prevent one from ending up in a burnout or lacking in study success. The aim of this study is to explore 1) how the identified underlying stressors are related to perceived stress and mental wellbeing, 2) to what extent the association between underlying stressors and mental wellbeing are mediated through perceived stress and 3) which factors can buffer or reinforce the association between perceived stress and mental wellbeing among university students.

## Method

### Design

A cross-sectional online survey study was conducted between November 16, 2020, and January 18, 2021. Ethical clearance was obtained under the license of the master’s program Health Education and Promotion at Maastricht University (number *i6217401)*.

### Sample

Prospective participants were students from the HZ University of Applied Sciences in The Netherlands. All students (n = 4800) could participate in the study. No exclusion criteria were used. To have sufficient power for the mediation and moderation analyses, at least 356 students had to participate in the study.

### Recruitment and procedure

Recruitment materials were designed and distributed by second-year students of International Business and Management (IBM) at HZ, who acted as data-collection assistants (DCA’s). This method of data collection has been used to promote the research in a more personalized way and, as a result, to increase the number of respondents. The DCA’s were divided into small groups, each responsible for data collection in one of the Bachelor study programs at HZ. The DCA’s developed interactive videos in which they explained the purpose and the value of the study. The video was then shown to the students in the Bachelor study programs, with DCA’s providing additional explanations. In addition, all students received an information letter by e-mail geared to the language used in the study program, Dutch or English. The letter contained general information about the purpose of the study, the time it took to participate, the consideration, privacy conditions (including anonymity) and the option to withdraw at any time without consequences. As a token of appreciation, participants had a chance to win an iPhone SE 2020 and ten EUR 15,—gift vouchers from Bol.com. Finally, the letter contained a digital reference to the questionnaire, in both Dutch and English. Students wanted to participate were automatically directed to an information page in which they had to give informed consent. Students could only participate in the study if they gave informed consent.

### Questionnaire and measures

The online questionnaire was provided through the online survey program Survalyzer. Both, a Dutch and an English version, were offered and students could choose the preferred language. Translations of the questionnaires were taken from recently conducted studies and checked by several independent researchers. Before the questionnaire was distributed to the entire population, it was pre-tested by 60 International Business students. After that, several small adjustments were made, such as a larger font. The measured concepts were mental wellbeing, perceived stress, coping style, self-esteem, loneliness, personality, academic pressure, family pressure, financial pressure and side-activity pressure and demographic factors. For measurement of each concept existing validated questionnaires were used. Dutch language versions of the instruments that had been used in previous studies were used, after checking by several independent researchers for accuracy of translations. Completing the questionnaire took on average 15 minutes. Table 1 lists studied concepts with number of items, range, and internal consistency.

**Table 1:** Study variables with measures and internal consistency.

Concept	Questionnaire	Scale	Items	Cronbach’s Alpha
**Mental wellbeing**	Warwick-Edinburgh Mental Wellbeing Scale [3].	14–70	14	α 0.905
**Perceived stress**	Perceived Stress Scale [14].	0–40	10	α 0.854
**Coping style**	Brief COPE [38].	12–48	24	α 0.770
**Self-esteem**	Rosenberg Self Esteem Scale [39].	0–30	10	α 0.884
**Loneliness**	CEL Loneliness Scale [40].	3–12	3	α 0.856
**Personality**	Ten Item Personality Measure (TIPI) [41].	1–7	10	α 0.536
**Academic pressure**	Perceptions of Academic Stress [19].	1–5	18	α 0.891
**Financial pressure**	Adolescent Stress Questionnaire [42].	1–5	6	α 0.871
**Family pressure**		1–5	6	α 0.775
**Side-activity pressure**		1–5	6	α 0.915

Mental wellbeing was measured with the Warwick-Edinburgh Mental Wellbeing scale [3]. This questionnaire consisted of fourteen items focusing on psychological functioning, life satisfaction, and ability to develop and maintain mutually beneficial relationships. The answering format is a five-point Likert scale from "none of the time" to "all of the time". An example question is ‘*In the past two weeks*, *I’ve been feeling optimistic about the future’*. The questionnaire has successfully been applied in several student surveys and was found to be valid [43]. When the internal consistency was found to be sufficient, a composite score was calculated based on a sum with a range of 14 to 70. A higher score represents better mental wellbeing. No cut-off score was available, however previous research among students in Scotland had an average score of 51.8 [44].

Perceived stress was measured with the Perceived Stress Scale [14]. This is a validated ten item questionnaire that assesses the level of stress people perceive in different situations. Items in this questionnaire addressed feelings and thoughts with focus on the past month. An example question is ‘*In the last month*, *how often have you been upset because of something that happened unexpectedly*?*’* The answering format was a five-point Likert scale from "never" to "always". When the internal consistency was found to be sufficient, the scale score was calculated based on a sum using a range between 0 and 40. Based on the score a categorization can be made in low perceived stress 0–13, moderate perceived stress 14–26 or a high amount of perceived stress 27–40.

Coping style was measured with the Brief COPE, a 28-item validated questionnaire of strategies used to deal with or regulate cognitions in response to stressors [38]. Twelve items relate to approach coping and twelve items to avoidant coping. Participants were asked to reflect on life events. An example question is ‘*I’ve been taking action to try to make the situation better’*. The other four items dealt with humor and religion and were not part of approach or avoidant coping. To keep the questionnaire as compact as possible, it was decided to remove these items. The answering format was a four-point Likert scale from ‘I haven’t been doing this at all’ till ‘I have been doing this a lot’. When the internal consistency was sufficient, the scores of both coping styles were determined based on a sum score with a range of 12 to 48.

Self-esteem was measured with the Rosenberg Self Esteem Scale [39], a ten-item validated questionnaire that provides insight into participant’s self-esteem. An example question is ‘*On the whole*, *I am satisfied with myself’*. The answering format was a four-point Likert scale from "strongly disagree" to “strongly agree”. When the internal consistency was found to be sufficient, the scale score was calculated based on a sum score with a range of 0 to 30.

Loneliness was measured with the CEL Loneliness scale [40], a three-item validated questionnaire measuring loneliness. An example question is *‘I am content with my friendships and relationships’*. The answering format was a four-point Likert scale from "strongly disagree" to “strongly agree”. When the internal consistency was found to be sufficient, the scale score was calculated with a sum score ranged 3 to 12.

Personality was measured with the Ten Item Personality measure (TIPI), a ten-item validated questionnaire based on the Big Five personality dimensions [41]. The personality traits extraversion, agreeableness, openness to experiences, conscientiousness and emotional stability were measured. An example question is *‘I see myself as extraverted*, *enthusiastic”*. Due to a negative internal consistency of the items measuring agreeableness, they were separated into two personalities: critical and sympathetic. The answering format was a seven-point Likert scale from “disagree strongly" to "agree strongly”. By using a mean score, the score on the personality trait was calculated with a range of 1 to 7.

Academic pressure was measured with the validated 18 item Perceptions of Academic Stress questionnaire [19]. The questionnaire was developed to measure academic pressure among students and focused on: pressures to perform, perceptions of workload and examination, self-perceptions, and time restraints. An example question is *‘I am confident that I will be a successful student’*. The answering format was a five-point Likert scale from “disagree strongly" to "agree strongly”. When the internal consistency was found to be sufficient, the scale score was calculated with a mean score ranged 1 to 5.

Family pressure, financial pressure and side-activity pressure were measured with the Adolescent Stress Questionnaire [42]. This is a validated 56-item questionnaire designed to determine stress categories in adolescents, of which 18 items were used in this study. The components of the questionnaire are stress of home life, stress of school performance, stress of school attendance, stress of romantic relationships, stress of peer pressure, stress of teacher interaction, stress of future uncertainty, stress of school/leisure conflict and stress of financial pressure. An example question is *‘Not enough money to buy the things you need’*. Only the categories stress of home life, stress of school/leisure conflict and stress of financial pressure were used. The answering format was a five-point Likert scale from “disagree strongly" to "agree strongly”. When the internal consistency was found to be sufficient, the scale score was calculated with a mean score ranged 1 to 5.

Finally, the following demographic characteristics were measured: gender, age, educational form, academic year, educational program, educational level, living situation, student loan and occupation. Gender had three categories: man, woman and gender-fluid. Age was open and filled in with two digits. Educational form had two options: full-time or part-time. The academic year had the options: 1, 2, 3, 4 and 5+. Educational program made it possible to select one program from all offered programs. Educational level had three options: associate degree, bachelor’s degree, and master’s degree. Living situation was subdivided into living independently, living with parents/caregivers or another situation. Student loan and occupation were both binary questions (yes or no).

### Analysis

Data analysis was performed with the statistics program SPSS, version 27. Descriptive statistics were used to describe the study population and to gain insight into scores of the main concepts. Next, we looked at the associations between variables using bivariate correlation analysis between the main concepts of the conceptual model (Fig 1). To better understand the direct relations between variables, simple linear regression analysis was performed for the relation between perceived stress and mental wellbeing, and between underlying stressors and mental wellbeing. Multiple linear regression analysis was performed to gain insight in the association between underlying stressors as independent variables and perceived stress as dependent variable. To assess whether perceived stress mediates the relation between the underlying stressors and mental wellbeing a mediation analysis was performed, using Hayes PROCESS tool mediation model 4. To assess whether loneliness, personality, self-esteem and coping style moderated the relation between perceived stress and mental wellbeing a series of moderation analyses was performed using Hayes PROCESS tool moderation model 1. When there was significant interaction, simple slopes analyses were performed. Three values of the moderator variable were presented: low (-1 SD), medium (Mean) and high (+1 SD). A significance level of *p* < 0.1 was used in the PROCESS analyses.

## Results

### Participants

Of the 1855 students opened the online questionnaire. About half dropped out or did not give informed consent. A total of 875completed the study. The majority of the participants were women 62.3% (n = 545) (Table 2). The vast majority of participants studied full-time 91.2% (n = 798). The mean age was 21.6 years (*SD* 5.4). Students from all academic years were represented in the study, but the majority were from years one 29% (n = 254) and two 33.6% (n = 294). Among the participant, 92.5% (n = 809) was in a bachelor’s program. Most participants lived with their parents or caregivers, 59.4% (n = 520). Finally, 72.2% (n = 632) had a job alongside the study and 35.3% (n = 309) had a student loan.

**Table 2:** Demographic characteristics of participants (n = 875).

	Category	*n*	%*	M(SD)
**Gender**	*Male*	*326*	37,3	**-**
	*Female*	*545*	62,3	
	*Gender-fluid*	*4*	0,5	
**Age**			-	*21*,*6(5*,*4)*
**Educational form**	*Full-time*	*798*	91,2	**-**
	*Part-time*	*77*	8,8	
**Study year**	*1*	*254*	29,0	**-**
	*2*	*294*	33,6	
	*3*	*162*	18,5	
	*4*	*150*	17,1	
	*5+*	*15*	1,7	
**Educational level**	*Associate Degree*	*57*	6,5	**-**
	*Bachelor*	*809*	92,5	
	*Master*	*9*	1,0	
**Living situation**	*Independent*	*312*	35,7	**-**
	*Caregivers*	*520*	59,4	
	*Other*	*43*	4,9	
**Side job**		*632*	72,2	**-**
**Study loan**		*309*	35,3	**-**

*Percentages are rounded to a decimal.

### Mean scores of study variables

Table 3 presents the mean scores of all study variables. Mental wellbeing had a mean score of 48.7 (Standard deviation (*SD)* 8.4; scale range 14–70), indicating an average score. The mean score for perceived stress was 19.7 (*SD* 6.6) on the scale ranging from 0 to 40. A total of 15.3% experienced a high amount of stress, 65.8% moderate and 18.9% a low amount of stress. On average, approach coping 32.6 (*SD* 5.9) was more dominant than avoidant coping 23.8 (*SD* 4.7) on a scale ranging from 12 to 48. Self-esteem scored medium to high with a mean score of 19.2 (*SD* 5.2). Loneliness among participants scored low to average with a mean score of 5.6 (*SD* 2.0) on a scale ranging from 3 to 12. All underlying stressors had a scale range from 1 to 5. Side-activity pressure scored highest with a mean score of 3.2 (*SD* 1.0). This was followed by financial pressure with a mean score of 2.8 (*SD* 1.0), academic pressure with a mean score of 2.6 (*SD* 0.6) and eventually family pressure with a mean score of 2.2 (*SD* 0.9). Finally, six types of personality were measured with a scale range from 1 to 7. The most dominant personality was sympathetic with a mean score of 5.8 (*SD* 1.1). This was followed by conscientiousness with a mean score of 5.4 (*SD* 1.2), openness to experiences 5.2 (*SD* 1.1), emotional stability 4.6 (*SD* 1.5) and extraversion 4.4 (*SD* 1.5). The personality of criticality was less present and had a mean score of 2.5 (*SD* 1.3).

**Table 3:** Correlation matrix and mean scores of all study variables.

STUDY VARIABLES	1	2	3	4	5	6	7	8	9	10	11	12	13	14	15	16	*M(SD)*
1. MENTAL WELLBEING	-																*48*,*7(8*,*4)*
2. PERCEIVED STRESS	**-,667** **	-															*19*,*7(6*,*6)*
3. APPROACH COPING	**,339** **	**-,160** **	-														*32*,*6(5*,*9)*
4. AVOIDANT COPING	**-,384** **	**,493** **	**,147** **	*-*													*23*,*8(4*,*7)*
5. SELF-ESTEEM	**,641** **	**-,547** **	**,261** **	**-,474** **	*-*												*19*,*2(5*,*2)*
6. LONELINESS	**-,452** **	**,363** **	**-,293** **	**,212** **	**-,445** **	*-*											*5*,*6(2*,*0)*
7. ACADEMIC PRESSURE	**-,538** **	**,589** **	**-,199** **	**,391** **	**-,556** **	**,319** **	*-*										*2*,*6(0*,*6)*
8. FAMILY PRESSURE	**-,359** **	**,374** **	**-,128** **	**,357** **	**-,395** **	**,311** **	**,460** **	*-*									*2*,*2(0*,*9)*
9. SIDE-ACTIVITY PRESSURE	**-,370** **	**,530** **	**-,032**	**,287** **	**-,291** **	**,259** **	**,531** **	**,378** **	*-*								*3*,*2(1*,*0)*
10. FINANCIAL PRESSURE	**-,245** **	**,331** **	**,001**	**,261** **	**-,252** **	**,199** **	**,277** **	**,456** **	**,439** **	*-*							*2*,*8(1*,*0)*
11. EXTRAVERSION	**,270** **	**-,121** **	**,238** **	**-,035**	**,337** **	**-,328** **	**-,200** **	**-,131** **	**-,052**	**-,020**	*-*						*4*,*4(1*,*5)*
12. CRITICAL	**-,158** **	**,029**	**-,120** **	**-,018**	**-,139** **	**,119** **	**,115** **	**,049**	**-,009**	**,023**	**-,176** **	*-*					*2*,*5(1*,*3)*
13. SYMPATHETIC	**,166** **	**-,052**	**,217** **	**-,068** *	**,180** **	**-,243** **	**-,104** **	**-,181** **	**-,026**	**,005**	**,177** **	**-,026**	*-*				*5*,*8(1*,*1)*
14. CONSCIENTIOUSNESS	**,258** **	**-,207** **	**,214** **	**-,255** **	**,294** **	**-,173** **	**-,306** **	**-,195** **	**-,067** *	**-,112** **	**,104** **	**-,162** **	**,196** **	*-*			*5*,*4(1*,*2)*
15. EMOTIONAL STABILITY	**,497** **	**-,550** **	**,074** *	**-,448** **	**,557** **	**-,310** **	**-,410** **	**-,324** **	**-,291** **	**-,229** **	**,130** **	**-,018**	**,119** **	**,201** **	*-*		*4*,*6(1*,*5)*
16. OPENNESS TO EXPERIENCES	**,191** **	**-,076** *	**,218** **	**-,045**	**,249** **	**-,175** **	**-,223** **	**-,095** **	**-,058**	**,002**	**,242** **	**-,087** *	**,200** **	**,152** **	**,150** **	*-*	*5*,*2(1*,*1)*

** Correlation is significant at the 0.01 level,

* Correlation is significant at the 0.05 level.

### Bivariate correlations between study variables

There was a strong negative correlation between perceived stress and mental wellbeing (Pearson’s correlation (*r)* = -.67; *p* < .01) (Table 3). Also, the four categories of stressors had a negative correlation with mental wellbeing, with academic pressure having the strongest negative correlation (*r* = -.54; *p* < .01). All stressors had a positive correlation with each other and with perceived stress. There was a strong positive correlation between self-esteem and mental wellbeing (*r* = .64; *p* < .01) and a negative correlation between self-esteem and perceived stress (*r* = -.55; *p* < .01). Loneliness was negatively correlated with mental wellbeing (*r* = -.45; *p* < .01) and positively with perceived stress (*r* = .36; *p* < .01). Finally, the personality trait, emotional stability had a moderate to strong correlation with mental wellbeing (*r* = .50; *p* < .01) and perceived stress (*r* = -.55; *p* < .01).

### Simple and multiple linear regression

Perceived stress was significantly associated with mental wellbeing (*b* = -0.848, p < 0.001) and explained 45% of the variance in mental wellbeing. Academic pressure, family pressure, side-activity pressure and financial pressure were significantly associated with perceived stress and explained 42% of the variance in perceived stress. Academic pressure (*b* = 4.736, *p* < 0.001) and side-activity pressure (*b* = 1.676, *p* < 0.001) had a strong positive significant association with perceived stress. Financial pressure (*b* = 0.525, *p* < 0.01) and family pressure (*b* = .373, *p* = 0.09) had a moderate to small association with perceived stress.

### Mediation through perceived stress

**Fig 2:**
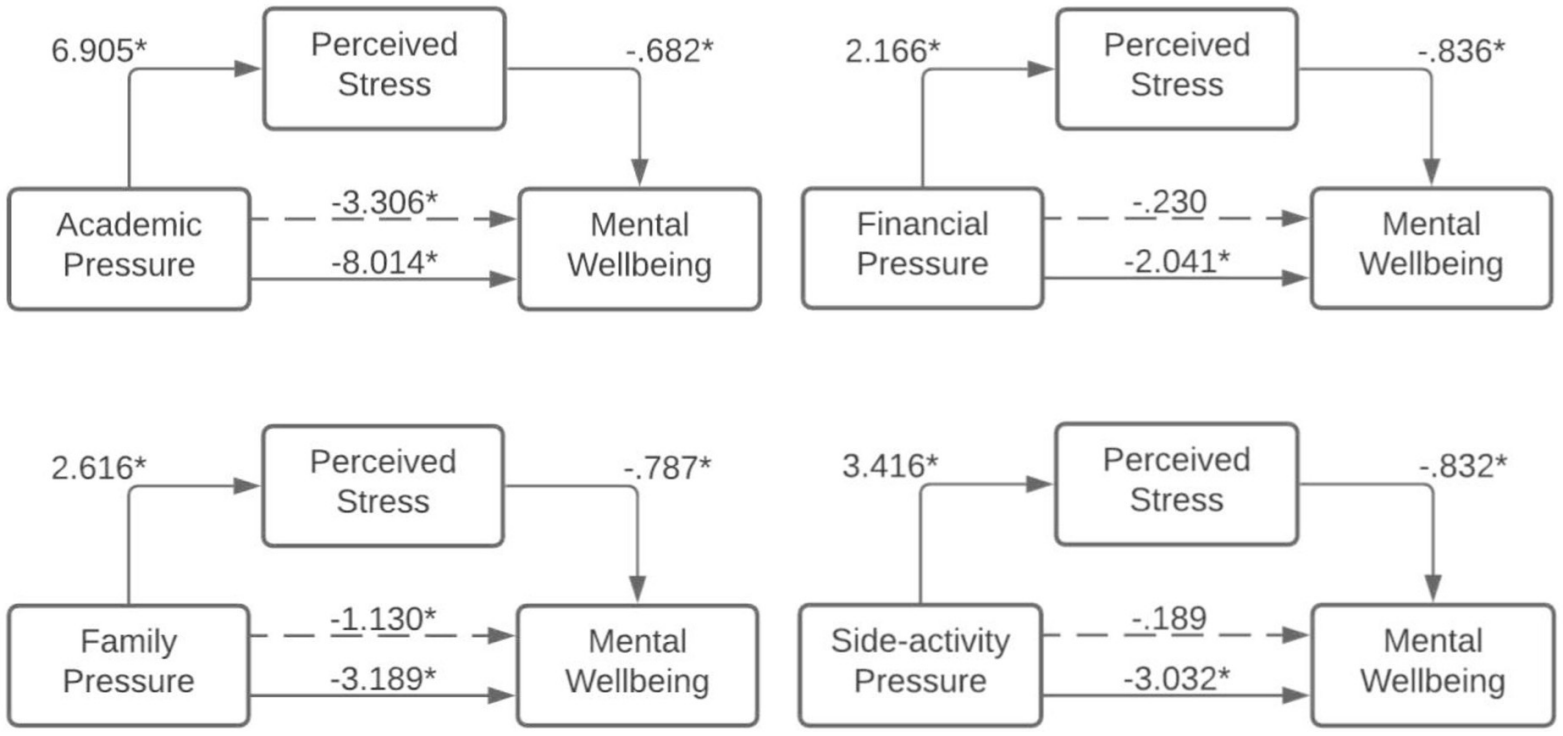
Mediation models of perceived stress between underlying stressors and mental wellbeing showing the total effect (solid line, c) and direct effect after mediation (broken line c’). * p is significant at the <0.01 level.

Fig 2 shows four mediation models, where the independent variables are the underlying stressors. The mediator is perceived stress, and the dependent variable is mental wellbeing. Each mediation model describes four relations: from independent variable to the mediator, from mediator to the dependent variable, the total effect of the independent variable on the dependent variable (solid line), and the direct effect of the independent variable on the dependent variable in the mediation model (broken line). As Fig 2 illustrates, all underlying stressors had a significant association with mental wellbeing (solid line from stressor to mental wellbeing). The strongest association was found for academic pressure, followed by family pressure, side-activity pressure, and financial pressure. For all stressors, there was a significant mediation by perceived stress. Complete mediation took place in the models of financial pressure and side-activity pressure, as the direct effect disappeared after presence of the mediator (dashed line). The indirect effect of financial pressure on mental wellbeing was significant [Effect = -1.811; CI (-2.21, -1.44)]. This also applied to the mediation model of side-activity pressure [Effect = -2.843; CI (-3.24, -2.47)]. Partial mediation took place in the models of academic pressure and family pressure, with a significant direct effect remaining after the presence of the mediator (broken line from stressor to mental wellbeing). However, a significant indirect effect also appeared in these models, considering academic pressure [Effect = -4.708; CI (-5.47, -3.99)] and family pressure [Effect = -2.060; CI (-2.45, -1.68)].

### Moderation between perceived stress and mental wellbeing

**Fig 3:**
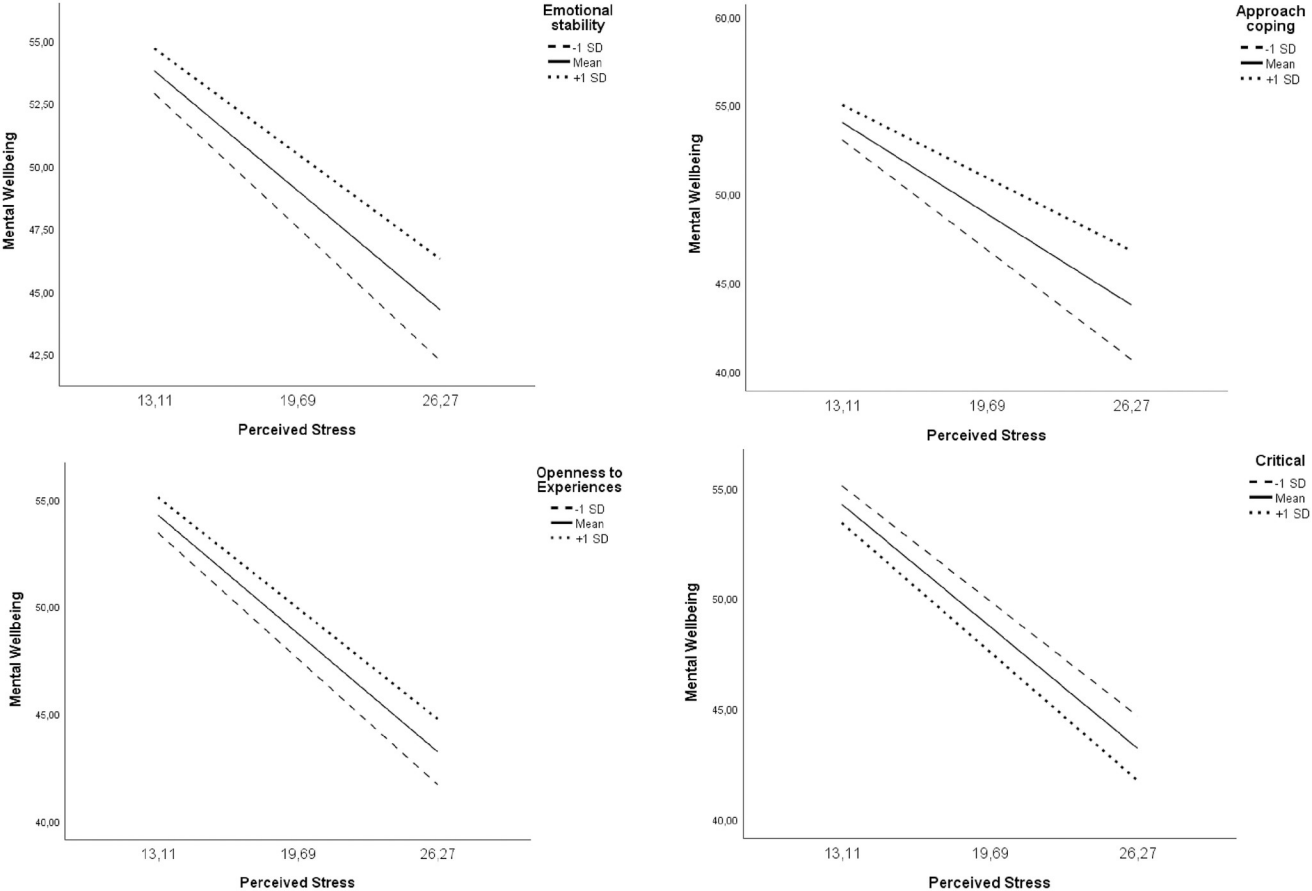
Simple slopes of perceived stress interacting with mental wellbeing for 1 SD below the mean, the mean, and 1 SD above the mean of emotional stability, approach coping, openness to experiences and critical.

A significant interaction with perceived stress was found for emotional stability, approach coping and the personality traits openness to experiences and critical (Table 4). Subsequent simple slopes analyses show the conditional effects (CE) for the moderator variable at -1 SD, M and +1 SD (Fig 3). The association between perceived stress and mental wellbeing was stronger at low levels of emotional stability (CE = -.81; *p* = < .01) than at high levels (CE = -.64; *p* = < .01). This was also seen in approach coping. At low levels there was a stronger association (CE = -.93; *p* = < .01) than at high levels (CE = -.64; *p* = < .01). Similar findings were made in the personality trait, openness to experiences. At low levels of openness to experiences (CE = -.89; *p* = < .01), the association was stronger than at high levels (CE = -.79; *p* = < .01). Finally, the personality trait, critical showed opposite results. At low levels of critical (CE = -.79; *p* = < .01), the association was less strong than at high levels (CE = -.89; *p* = < .01).

**Table 4:** Personal resources and characteristics tested for moderation between perceived stress and mental wellbeing.

Moderator variables	B	SE	t	p
**Emotional stability**	,058	,020	2,928	<,01**
**Approach coping**	,027	,005	5,406	<,01**
**Openness to experiences**	,049	,029	1,694	,09*
**Critical**	-,035	,021	-1,663	,09*
**Self-Esteem**	,008	,005	1,599	,11
**Conscientiousness**	,043	,027	1,586	,11
**Extraversion**	,026	,019	1,346	,18
**Avoidant coping**	-,007	,007	-1,058	,29
**Loneliness**	-,008	,014	-,578	,56
**Sympathetic**	,011	,028	,400	,69

** p is significant at the <0.01 level,

* p is significant at the <0.1 level.

Presentation of Hayes PROCESS moderation analyzes with the interaction term (B), Standard error (SE), t-value (t) and significance level (p).

## Discussion

The present study aimed to explore 1) how the identified underlying stressors are related to perceived stress and mental wellbeing, 2) to what extent the association between underlying stressors and mental wellbeing are mediated through perceived stress and 3) which factors can buffer or reinforce the association between perceived stress and mental wellbeing among university students. In this study, perceived stress had a strong negative association with mental wellbeing and 45% of the variance in mental wellbeing was explained by perceived stress. A strong association has also been found between the underlying stressors and perceived stress, explaining 42% of the variance in mental wellbeing. Of the underlying stressors, academic pressure had the strongest negative impact on mental wellbeing and side-activity pressure had the highest score compared to other underlying stressors. The impact of side-activity pressure and financial pressure on mental wellbeing was entirely mediated by perceived stress. Academic pressure and family pressure were partly mediated by perceived stress, but also had a direct effect on mental wellbeing in the mediation model. Emotional stability, approach coping, openness to experiences and being critical showed a positive moderation effect on the relation between perceived stress and mental wellbeing. Emotional stability and approach coping most strongly buffered the impact of perceived stress on mental wellbeing.

The strong association between perceived stress and mental wellbeing found in this study illuminates that students with increased levels of stress are at high risk of impaired mental wellbeing. These findings are in line with those of previous studies that mainly showed that students with high stress levels had an increased risk of burnout-related complaints [4,5]. A strong association between perceived stress and mental wellbeing was also found in a study among nursing students [16]. However, the association found in our study was even stronger. This is striking, as it has previously been shown that in particular medical students have increased stress levels [45]. This may be explained by the low level of mental wellbeing in the current sample. Even though no cut-off score is available for the Warwick Edinburgh Mental Wellbeing Scale, the scores in this study were considerably lower as reported in previous student research [44]. The possible explanation is that this study has been conducted during the time that students have been confronted with the impact of the corona pandemic.

Perceived stress was largely explained by the underlying stressors, with academic stress having the strongest association. Nevertheless, all underlying stressors had a significant impact on mental wellbeing. This was largely mediated by perceived stress, but strikingly, a significant direct effect for academic pressure and family pressure also persisted in the mediation model. The impact of these underlying stressors on mental wellbeing was therefore partly external to perceived stress. Academic pressure had by far the greatest impact on mental wellbeing what is in line with previous research, where it turned out to be a decisive determinant [1,16,20]. It seems that reducing academic pressure could lead to less negative impact on mental wellbeing. Although academic pressure was most strongly related to mental wellbeing in the data analysis, the students reported side-activity pressure as the most stressful. Indeed, it seems that having a part-time job in combination with academic commitments implies a lack of leisure time, which is perceived as very negative [24]. One explanation that this type of stress is less strongly associated with mental wellbeing is that side activities may also contain pleasurable elements and that people are usually free in making choices for side activities. On the other hand, students feel that academic pressure belongs in a study and are less likely to label this as most excessive. Academic pressure is complicated and consists of different items e.g., pressure to perform, perceptions of workload, examination, self-perceptions, and time restraints [19]. Following the vision of Dopmeijer and Othman et al. on the importance of early identification of risk factors that put mental wellbeing at risk, it is important to further investigate or intervene on underlying stressors [10,12]. Universities have a large share in the way in which students experience stress, especially with regard to academic pressure, which in several studies has a strong negative impact on mental wellbeing [20,21].

In addition, this study focused on protective factors for the impact of perceived stress on mental wellbeing. The coping style used by a student directly influences the way perceived stress affects mental wellbeing. Applying approach coping buffers this impact. Similar findings have been made in previous studies, using the corresponding concept of problem-focused coping [33–35]. Besides coping style, the degree of emotional stability also appeared to have an impact on the way in which stress affects mental wellbeing. This may well be related to the use of coping. The moment a student is emotionally unstable, it seems more difficult to cope with adversity and stress, resulting in a deteriorated mental wellbeing [46]. Several personality traits, including emotional stability, have been identified as moderators between perceived stress and mental wellbeing [31,32]. It seems that personality traits play a decisive role in coping, perceived stress and mental wellbeing. To enhance the use of beneficial coping styles and reduce the impact of stress on mental wellbeing, it is important for universities to integrate the teaching of an approach coping style in curricula. In addition, emotional stability can be improved by educational techniques increasing the resilience of students. Coping interventions, such as the *’Resilience and Coping Intervention (RCI)*’ are applicable at the university or other school settings [47]. RCI is a youth group intervention designed to help participants identify thoughts, feelings, and coping strategies associated with problems following a stressful event or with everyday stressors. Houston et al. tested the RCI among college students, where after three 45-minute sessions the intervention group already had less stress and more hope and resilience compared to the control group [48]. These are promising results and would fit well in the current context. In summary, promoting mental health competencies, such as developing an approach coping style, will help to process perceived stress and to enhance mental wellbeing [10].

In this study loneliness and self-esteem did not moderate the relationship between perceived stress and mental wellbeing. As self-esteem and loneliness are both strongly correlated with perceived stress and mental wellbeing, these are potentially important determinants to take into account when explaining mental wellbeing. However, it was expected that both self-esteem and loneliness would influence or moderate the relationship between perceived stress and mental wellbeing. This expectation was outlined because previous research showed that a high level of self-esteem was associated with strong academic performance [26]. Loneliness was found to have a significant negative association with perceived stress and mental wellbeing [30].

### Limitations

The study was conducted in a cross-sectional design, which prevents making inferences on causal associations and on the direction of associations. It is not known whether an increased level of perceived stress precedes a deteriorated mental wellbeing or whether a deteriorated mental wellbeing leads to an increased level of perceived stress. Furthermore, the study was conducted at a single university of applied sciences. Results may differ for a different sample, for example in a different region or at a university of a different size. Data collection took place during the corona pandemic, so students may have had a different perception of stress. However, this study was not about the amount of stress, but the relation between stressors and mental wellbeing. Finally, it is known that certain demographic characteristics lead to an increased stress level (e.g., gender). In this study design, no attention was paid to distinction in demographic characteristics.

## Conclusion & recommendations

Perceived stress has a strong negative association with mental wellbeing. The underlying stressors investigated in this study were strongly related to perceived stress, and all had a negative association with mental wellbeing. Academic pressure, the most influential stressor, and family pressure had a direct impact on mental wellbeing that is not explained by perceived stress. Approach coping and emotional stability buffer the impact of perceived stress on mental wellbeing. To protect the mental wellbeing of students, it is urgent to suppress perceived stress and make students more resilient. Academic pressure must be reduced to lower students’ perceived stress. Additionally, facilitating students in mental health competences by teaching approach related coping styles and resilience skills. Further research is needed, as it is unclear why academic pressure and family pressure, in addition to mediation with perceived stress, also have a direct impact on mental wellbeing. A longitudinal study will provide insight into decisive underlying factors and explain more thoroughly the relationship between perceived stress and mental wellbeing.

## Supporting information

S1 Dataset (PDF)
