# Supplementary material for: How stress-related factors affect mental wellbeing of university students A cross-sectional study to explore the associations between stressors, perceived stress, and mental wellbeing
Source: PLoS One. 2022 Nov 7;17(11):e0275925. doi: 10.1371/journal.pone.0275925 (PMC9639818; doi:10.1371/journal.pone.0275925)
Supplement: S1 Dataset — (PDF) [file pone.0275925.s001.pdf]

Subject	Gender	Age	Year	Mental Wellbeing	Perceived Stress	Academic Stress	Financial Stress	Family Stress	Sideactivity Stress	Avoidance Coping	Approach Coping	Extraversion	Agreeableness	Conscientiousness	Emotionalstability	Openness	SelfEsteem	Loneliness
1	Male	18	1	48	20	2,89	2,67	2	3	21	24	7	6	10	13	9	2,9	8
2	Male	18	1	37	26	3,17	2,5	2,5	1,6	27	30	8	11	7	7	12	2,2	6
3	Male	21	4	53	15	2,39	4,17	4	3,8	19	39	7	8	13	13	12	3,7	7
4	Male	18	1	38	29	3,67	4,33	5	3,4	24	33	4	8	9	5	6	1,9	7
5	Male	21	3	41	26	3,33	3,5	3,75	3,2	25	31	14	9	7	10	12	3	11
6	Male	21	3	59	2	1	1,67	1	1	19	40	9	14	14	14	13	4	10
7	Female	22	3	26	34	2,83	4,5	3	3,4	37	30	10	10	8	4	11	1,8	6
8	Male	32	4	51	12	2,33	1	1,5	2,8	22	37	6	6	12	11	9	3,2	12
9	Female	20	2	49	25	3,44	4,17	3,5	3,4	21	38	12	6	12	9	12	2,4	12
10	Male	21	4	46	20	2,5	3,17	1,75	3	19	19	5	8	9	13	7	2,8	8
11	Male	22	3	53	15	2,06	3,33	1,25	2,8	20	35	6	11	14	10	10	3,1	10
12	Female	20	4	49	28	2,44	4	1,75	4,8	18	39	10	10	11	5	14	3	10
13	Female	20	1	47	18	3,56	1,83	4	3	26	45	5	7	9	6	13	2,5	9
14	Male	23	4	46	18	2,33	1	1,5	1,6	18	31	12	9	11	10	12	3,3	12
15	Male	18	2	50	14	2,94	2,67	1,75	1,6	18	33	10	11	7	13	7	3,5	11
16	Female	19	1	49	16	2,94	3,33	2,75	4	20	32	2	11	14	10	12	3,3	9
17	Male	19	1	57	16	1,94	1,67	1,25	1,6	21	35	12	9	11	8	11	2,9	12
18	Female	21	4	40	26	2,78	3,5	3,25	4,2	28	29	8	6	11	6	10	2,5	7
19	Male	19	2	46	11	2,28	3,17	1	2	18	24	10	4	9	12	10	3	6
20	Female	21	2	54	20	2,28	2,5	1	1,8	18	24	13	8	13	12	12	3,6	12
21	Male	23	2	42	27	3,28	2,33	2	2,8	24	39	10	9	12	9	11	3	12
22	Female	22	3	44	20	3,17	3,67	3,25	4	24	37	9	9	12	11	12	2,3	12
23	Male	23	2	55	9	2,11	2,33	1	1,6	15	18	4	8	8	12	11	3,2	12
24	Male	24	3	41	21	2,94	1,67	1,5	1,4	23	25	4	8	10	12	8	1,8	9
25	Male	24	3	37	8	2,94	2,5	1,5	1,2	22	30	4	6	13	13	9	1,9	9
26	Female	22	4	53	17	2	2,5	1,75	2,2	24	41	11	10	13	7	11	2,5	12
27	Female	24	5+	42	25	3,17	3,33	3,75	2,2	29	23	3	9	8	6	7	2,1	7
28	Male	19	3	42	21	2,11	3,83	3,5	3	26	32	7	7	12	7	9	2	8
29	Male	22	4	55	16	3,06	2,5	2,75	2,4	36	36	7	8	9	7	8	2,5	9
30	Male	22	3	47	25	1,83	2,83	2,75	1,2	26	44	9	10	14	11	13	2,7	8
31	Female	21	3	48	30	2,78	3	1,5	3,4	26	36	14	8	13	6	14	3,3	9
32	Male	25	4	35	28	3	3	4	3,2	28	25	9	7	14	3	7	2,7	7
33	Female	23	4	62	13	2,83	1,17	1,75	2,8	20	38	8	8	13	7	14	3,8	12
34	Neutral	25	4	54	19	2,44	2,5	1,75	2,8	28	32	8	6	11	6	11	2,8	9
35	Female	24	1	46	12	2,33	3,83	1,75	3,4	20	30	7	9	11	11	10	3	11
36	Female	17	1	39	29	3,5	3,5	3,75	3,4	33	29	8	11	4	4	11	2,4	11
37	Male	20	2	46	27	3	1,67	1,75	3,6	26	45	11	12	13	13	13	3,6	8
38	Male	20	4	40	24	2,5	2,17	2,75	3,6	23	30	10	9	7	6	10	2,8	8
39	Female	18	1	48	11	1,89	1,83	2	2,2	18	28	11	13	11	12	13	3	9
40	Male	18	1	39	25	3,06	3,33	2,75	3,8	36	33	6	9	9	6	10	2	6
41	Male	18	2	56	14	1,94	1,67	1	1	15	22	13	8	12	13	14	4	12
42	Male	22	4	44	17	2,61	2	1,5	3	22	38	8	10	12	8	12	2,8	10
43	Male	17	1	52	19	2,83	2,33	1	3,4	23	27	9	8	10	10	7	3	8
44	Female	17	1	51	11	2,44	2,17	1	1,8	21	35	11	12	11	14	13	3,8	12
45	Male	21	4	52	20	3,22	1,33	1	1	24	27	10	13	9	11	7	2,5	12
46	Female	20	4	48	26	2,61	2,67	1,25	3,4	21	33	10	7	13	7	10	3,6	12
47	Female	19	2	42	21	2,94	3,83	1,75	4	31	26	5	9	9	10	13	2,7	9
48	Female	17	1	56	24	2,67	1,67	1,5	3,6	21	28	7	12	14	8	12	3,1	8
49	Male	21	3	41	21	2	2,5	2,25	2,4	25	26	13	8	12	8	12	2,3	8
50	Female	24	4	48	23	2,89	4	3,5	4,2	20	32	8	8	14	10	13	3,1	12
51	Female	17	1	47	13	2,61	3,17	2,75	3,6	21	27	8	12	9	5	10	3,3	12
52	Male	18	1	51	8	2,67	3	1	2,8	20	28	6	9	12	13	11	2,8	9
53	Male	20	4	48	19	2,67	2,83	2,25	2,2	24	30	3	8	8	9	5	2,8	9
54	Male	22	1	53	19	2,17	1,83	2,25	3,8	18	22	4	5	11	8	8	3,9	7
55	Male	19	1	55	13	2,39	1,67	1,75	2	27	32	11	8	14	10	8	3,5	12
56	Male	25	3	58	20	1,44	1	1	1,6	28	35	10	8	12	13	12	3,7	12
57	Female	23	1	64	13	1,78	2	2	2	22	39	13	9	11	12	14	3,8	12
58	Female	19	1	35	24	3,17	3	3	2,6	28	30	5	8	8	6	12	1,8	8
59	Male	19	2	42	18	3,17	2,33	3	3,6	22	32	5	11	6	8	10	2,6	8
60	Female	19	2	62	15	1,67	3,17	1,5	1,4	21	35	12	7	10	7	11	3,8	12
61	Male	19	1	54	12	2,72	2,5	3	1,8	22	36	13	9	13	11	13	3,5	9
62	Male	22	3	55	18	1,56	3,33	1,5	3,6	21	35	8	7	13	10	10	3,1	7

63	Male	21	1	51	18	3,11	1,83	2,5	2,8	17	23	11	10	7	12	9	3,2	9
64	Male	20	3	45	23	2,11	1,17	2,25	3,4	18	32	6	8	14	13	9	3,3	6
65	Male	20	3	44	13	2,17	1,67	1,25	1,8	25	35	9	8	12	11	11	3,2	12
66	Female	20	1	60	8	2,06	3	1,75	2,2	21	38	9	9	14	13	14	2,8	11
67	Male	20	2	51	12	2,22	2,5	2,5	2,4	23	38	9	7	9	13	13	3,2	12
68	Male	19	1	60	8	2,33	1,67	1,25	1,4	29	43	13	10	12	12	13	3,4	12
69	Male	20	4	58	11	1,78	1	1	1	17	29	11	7	10	11	11	3,4	10
70	Male	19	1	58	9	2,39	4	4	3,6	20	31	7	7	11	13	11	3	9
71	Male	21	4	53	14	2,61	2,5	3,5	2,2	25	39	3	8	11	10	11	3,6	12
72	Male	18	2	56	22	2,56	1,67	1,75	1,4	30	32	9	5	11	9	10	2,5	11
73	Male	18	1	55	11	2,44	1	1	1	17	27	8	9	12	11	10	3,7	11
74	Male	21	4	43	16	2,22	2,33	1	2,4	26	28	11	7	7	11	9	2,8	12
75	Female	29	4	34	21	2,83	1	1	1	19	30	4	11	12	6	9	2,9	9
76	Male	18	1	53	13	3	3,33	2,25	2,6	22	31	7	10	9	13	10	3,1	10
77	Male	21	3	43	24	1,56	2,67	2,5	2,8	21	35	4	8	14	8	11	3,2	9
78	Male	19	1	58	15	2,22	4	3	3,6	15	28	11	6	8	7	10	2,7	12
79	Female	19	2	51	24	2,44	3	2,5	1	33	41	8	10	11	5	13	2,8	11
80	Male	23	2	52	20	2,17	3,17	1	1,2	17	30	6	8	14	14	9	3,6	12
81	Male	19	1	56	19	2,94	2,67	2	3,4	29	38	9	9	14	11	12	2,5	9
82	Male	22	4	53	12	1,83	3,17	1,25	1	20	38	11	9	9	9	13	3,1	8
83	Male	21	4	37	19	2,94	2	2,5	3,4	22	24	9	9	10	12	9	2,7	8
84	Male	25	4	44	16	2,83	2	1,25	3,2	14	23	8	7	12	11	8	2,9	12
85	Male	24	4	56	20	2,11	2	2	2	16	30	8	8	8	8	8	3	9
86	Male	23	4	62	10	1,44	2,83	2	1,8	21	37	8	9	13	13	14	3,7	11
87	Male	33	4	31	30	2,56	1,67	2,75	3	25	24	8	5	8	3	8	2,3	3
88	Male	21	4	31	25	3,33	3,67	2	2,4	27	23	8	8	9	7	9	2,5	10
89	Female	22	4	35	31	3,28	4,33	4,25	3,8	29	33	6	9	7	2	12	2,2	9
90	Female	20	1	33	23	3,11	1	1,25	3	22	19	2	9	8	6	6	2,4	11
91	Male	20	2	50	17	2,28	2,5	2,5	2,2	21	30	7	9	13	12	11	2,9	9
92	Male	21	1	43	19	2,61	1,67	3	3	20	33	10	12	12	10	12	3,2	9
93	Male	21	4	55	20	3,22	4	3,5	3,8	36	31	10	8	9	10	9	2,7	9
94	Male	21	4	58	5	2,39	2,17	3	1,8	20	38	9	7	12	12	12	3,3	12
95	Male	17	1	59	11	2,17	1,17	1	1	20	36	9	9	12	12	11	3,6	11
96	Male	20	2	54	12	2,83	1,83	2,25	1,8	24	34	12	8	7	12	11	3,2	7
97	Male	22	4	55	10	1,56	1,17	1	1,2	22	33	9	8	14	13	10	3,2	11
98	Male	19	1	56	13	1,56	1	1,75	2,2	24	39	6	7	9	14	13	3,6	7
99	Male	20	4	55	13	2	1,17	2	1,4	18	28	7	6	13	7	6	3,1	9
100	Male	21	4	45	21	2,61	2,5	2	3	20	20	9	9	10	7	10	3	8
101	Male	21	4	47	20	2,83	4	4	4	28	33	11	7	9	8	9	2,7	9
102	Male	19	3	42	13	2,67	3,5	3	3,8	20	24	4	8	11	14	14	2,6	4
103	Male	22	3	30	32	3,17	3,5	3,25	3	23	22	2	9	7	7	9	2,2	3
104	Male	18	2	54	18	2,94	2,5	2,25	3,2	24	32	8	6	11	10	10	2,8	8
105	Male	22	4	50	19	2,56	2,33	2,75	3,4	27	40	4	10	10	12	10	3,3	8
106	Male	37	2	66	9	2,17	2	2	2,4	26	40	8	9	8	14	13	3,9	10
107	Male	20	1	57	17	2,39	3,67	4	1,8	31	39	12	8	10	7	8	2,5	8
108	Male	20	1	46	25	2,94	3,5	3,25	3,4	29	36	12	8	6	11	7	2,3	8
109	Male	22	1	46	18	2,33	3,33	1	3,6	21	30	7	14	12	11	10	3,1	7
110	Male	21	3	55	28	2,89	2,67	2,75	2,6	28	36	7	7	12	10	8	2,8	10
111	Female	19	3	40	24	3,5	4,17	3,75	5	25	39	9	5	11	8	8	2,5	8
112	Female	23	3	56	15	2,33	3,67	3	2,2	23	33	11	7	14	10	12	3,2	9
113	Male	19	3	48	21	2,28	2,5	2,25	3,8	22	34	7	7	9	8	9	3,4	9
114	Female	22	1	44	26	3,56	4	3	4	26	32	11	6	13	12	8	2,9	8
115	Male	19	1	53	15	2,39	2	2,5	2	25	30	7	4	11	12	14	2,9	9
116	Female	17	1	57	8	2,22	1,83	1,25	1,2	24	36	6	8	14	11	13	2,9	10
117	Male	19	3	45	22	3,17	2,5	1,5	1,8	20	34	14	8	9	12	11	3,2	9
118	Female	18	3	56	13	2,56	3,17	2	4,2	19	24	12	6	14	10	10	3,3	11
119	Male	20	1	53	14	1,89	3,5	2,5	2,6	23	28	12	7	13	8	11	3	6
120	Female	18	1	50	16	2,56	2,67	1,25	3,6	17	27	10	9	11	8	9	3	9
121	Female	27	4	41	23	2,89	3	2,5	4,2	33	35	10	7	9	9	12	2,9	10
122	Female	19	3	47	22	3	3,5	4,25	3,2	21	32	9	7	12	5	11	2,7	7
123	Female	18	3	66	12	1,61	1,17	1	1	23	44	14	7	10	8	14	3,5	12
124	Male	21	4	37	14	2,56	1,17	3,25	2,4	24	29	5	9	8	9	12	3,2	3
125	Female	19	3	55	27	2,56	2,67	2	4,4	23	40	8	8	8	7	12	2,9	7
126	Female	20	3	53	16	1,94	3	2,25	2,2	23	33	11	7	12	7	11	2,9	8

127	Male	20	1	49	21	2,17	3,17	2	4	19	42	10	8	10	12	14	3,7	11
128	Female	22	1	48	25	3,28	5	3,5	4,8	28	33	9	8	9	6	13	2,5	9
129	Female	22	4	55	13	2,61	3,5	2,5	2,6	21	33	4	8	12	12	8	3,3	9
130	Female	22	3	55	23	3,39	4,33	4,5	4	24	33	7	9	10	5	10	2,8	12
131	Female	17	1	46	26	3,17	2	3,5	3,2	28	30	8	8	12	3	11	2,2	8
132	Female	19	1	49	23	1,89	1	1,75	1	26	31	7	6	14	8	10	3,9	12
133	Male	17	1	62	6	2,33	3,33	2	3	23	33	10	9	9	14	12	3,7	12
134	Female	17	1	61	18	2,39	4	3,25	4	25	35	8	8	14	4	10	2,8	12
135	Female	18	1	46	16	3	2,5	2	3,8	29	32	9	9	9	10	10	3	9
136	Male	20	3	53	17	2,67	1,33	2	2,4	28	28	6	8	11	11	7	3	9
137	Male	26	3	45	15	2,78	2,67	3,25	2	23	29	9	12	8	10	11	2,9	7
138	Male	25	5+	48	20	2,44	4,67	2	3,4	24	38	11	7	13	11	7	3	6
139	Male	27	5+	46	26	2,39	4,17	3,5	4,2	28	28	12	8	10	12	12	3,1	10
140	Female	18	2	53	21	2,78	4,17	1	3	26	36	10	8	11	5	8	2,6	8
141	Male	22	3	52	12	2,22	1,83	2,75	2	32	33	11	8	9	12	10	2,8	12
142	Male	22	3	54	20	2,61	3,17	2,25	3,4	31	34	13	7	13	9	6	3	9
143	Male	23	3	56	22	3,33	2,33	2,75	2,8	34	34	10	7	11	9	12	3,5	9
144	Male	18	1	50	17	3,28	4	2,75	3,8	23	32	11	7	10	12	12	3,5	7
145	Male	19	3	44	17	3,44	1	2,75	2,6	19	32	4	8	8	7	8	2,6	8
146	Female	20	1	57	18	2,11	3,33	1,5	1	17	25	12	7	14	8	11	3,2	12
147	Male	18	3	53	19	1,94	2,17	2	1,4	30	32	12	6	9	9	10	3,3	9
148	Female	23	4	39	23	3,61	1	3,5	4	24	31	9	6	11	6	8	2	8
149	Male	19	3	56	23	1,72	2,5	1	2,2	19	21	11	8	14	14	11	4	11
150	Male	24	5+	50	19	3	4,33	3,75	3,8	28	35	7	9	6	6	11	2,5	11
151	Female	21	4	58	17	2,5	2,5	2,75	4,2	24	30	13	9	13	9	10	3,2	8
152	Female	20	2	54	24	2,33	3,67	3,5	2,8	27	34	11	8	13	7	11	3	12
153	Female	20	4	54	11	2,5	1,17	1	3	17	38	14	9	10	11	8	3,2	12
154	Male	16	1	57	8	1,89	2,83	1,5	2,6	22	38	9	8	10	12	9	3	8
155	Female	21	4	49	23	2,72	3,5	2	3,8	25	38	12	8	10	10	8	3,2	9
156	Male	21	3	53	20	1,83	1,17	2,75	2	18	28	9	7	10	11	9	2,8	9
157	Male	20	3	61	19	1,67	3	2,75	2,2	38	37	14	7	10	9	13	3,4	12
158	Female	19	3	49	16	3	3,83	3,75	3,8	23	31	10	7	11	7	8	2,7	12
159	Male	20	3	48	20	2,94	3,83	3,5	3,2	33	34	6	8	10	8	10	2,3	8
160	Male	22	3	54	10	1,78	1	1	1	14	20	12	7	13	14	12	4	12
161	Male	21	5+	39	23	3,11	3,17	3	4	27	22	9	10	7	10	9	2,8	9
162	Male	20	2	55	14	2,5	3,17	1,5	2,6	18	33	10	9	10	12	11	3,4	10
163	Female	20	3	54	12	2,17	3,83	3,25	2,2	22	35	12	8	10	12	12	3,4	11
164	Female	21	5+	46	27	3,11	1	3	4,2	28	35	8	8	12	6	9	2,5	10
165	Female	19	2	37	26	3,56	4	4	4	27	27	7	10	9	5	8	1,8	10
166	Female	18	2	47	22	2	2,17	3	3	30	35	14	8	9	6	13	2,7	8
167	Male	21	3	56	7	2,17	2	2	2	17	24	11	7	11	6	9	3,9	9
168	Female	37	3	56	10	1,89	2,5	1,5	2,2	20	36	13	9	14	14	13	4	10
169	Female	48	2	65	12	2,39	1	1	1	18	41	11	11	10	14	13	3,6	12
170	Female	17	1	53	25	2,33	2	2	3	25	36	12	8	11	8	11	3	12
171	Female	20	1	53	20	2,44	3,5	1	3	23	34	8	9	11	8	11	2,8	9
172	Female	17	1	55	12	2,22	4,17	3	3,2	22	33	7	11	8	12	10	3,1	9
173	Female	23	2	56	10	1,56	2,17	2,25	3,2	20	37	13	8	14	9	13	3,1	12
174	Female	23	1	46	17	2,67	4	2,75	4	24	28	10	8	11	10	9	3,4	6
175	Female	21	1	48	18	2,67	2,33	3,25	2,6	25	36	13	8	8	7	9	3,2	9
176	Male	31	3	48	21	2,5	1	1,5	2,4	24	38	9	9	14	12	11	3,3	10
177	Female	22	2	51	16	2,78	3	2,75	3,6	25	38	10	8	14	8	13	2,8	9
178	Female	24	3	52	24	3,17	2,17	3,75	4,8	26	35	13	9	14	6	12	3,3	12
179	Female	29	3	52	30	3,78	1,67	1	3,8	31	41	13	8	10	6	9	2,9	10
180	Female	28	2	51	16	1,78	1,5	1,5	3	24	31	7	9	14	13	13	3,5	11
181	Female	20	2	59	10	2,17	1,33	2	2	22	40	12	6	13	7	10	3,7	11
182	Female	17	1	41	17	2,67	3	2,25	3,4	28	30	7	9	10	7	7	2,2	11
183	Female	17	1	42	21	2,89	3,33	3,5	2,8	23	31	3	8	9	5	9	2,1	7
184	Female	27	2	41	28	2,83	4	2,5	5	25	33	12	7	9	4	12	3	12
185	Male	28	2	46	15	1,72	3,67	1	2,6	25	40	5	9	10	14	7	3,1	8
186	Male	47	1	54	13	1,44	2	1	1	18	36	14	9	12	13	12	3,5	10
187	Female	22	1	35	28	2,44	3,33	1,25	2,8	25	30	13	9	9	7	9	2,6	8
188	Female	22	3	43	25	2,89	4	1,25	2,6	23	32	8	8	13	10	8	2,7	9
189	Female	38	3	33	27	2,72	1,83	2,5	4,6	24	38	14	9	10	8	14	3,8	10
190	Female	26	2	52	26	2,22	2,83	1,75	3,6	20	41	9	9	13	11	14	3,2	9

191	Female	18	1	57	16	2,11	2	1,75	1,8	26	37	12	9	10	10	8	3,3	12
192	Female	28	4	49	24	2,83	2,83	1,5	5	25	36	12	8	9	13	9	3,4	12
193	Male	24	4	57	16	1,78	2,5	1	4	21	35	12	9	8	10	12	2,8	12
194	Female	48	2	51	27	2,67	3	1,75	4,6	25	37	12	7	8	7	14	2,5	6
195	Female	19	1	57	17	2,28	3,5	2	4	19	24	7	12	12	12	12	3	12
196	Female	19	1	64	7	2,06	3	1,5	3,4	20	38	13	8	11	11	10	3,1	9
197	Female	46	1	46	24	2,56	1	2,25	4	21	37	14	8	14	7	10	3	7
198	Female	35	1	56	16	2,28	2,67	3	3,4	23	33	14	9	14	10	14	3,2	11
199	Female	47	2	55	17	1,94	1	2,75	4,2	17	25	12	9	10	13	7	3,4	9
200	Female	23	2	42	26	2,94	4,83	3,75	4,8	26	39	3	6	12	6	10	2,4	8
201	Female	26	3	41	26	2,89	4,17	3	4	29	38	8	9	8	7	10	2,9	9
202	Female	20	1	50	18	3	4,5	2,5	3,6	27	36	14	10	14	10	13	2,6	8
203	Female	20	2	55	20	3	5	2,75	4	29	39	14	9	13	10	11	3,1	10
204	Female	20	2	50	16	2,67	2,67	2	4	20	34	4	6	14	14	13	3	8
205	Male	25	3	56	22	3	4	4	4	36	36	8	8	8	8	8	2,5	6
206	Female	36	1	51	20	2,44	1,83	1,5	2,4	23	42	5	7	14	9	11	1,6	7
207	Female	31	2	57	21	2,5	3,17	1	2,8	23	36	8	9	11	10	10	2,9	9
208	Female	37	2	47	31	2,83	1	2,25	5	23	30	12	9	8	8	10	2,7	10
209	Female	18	1	58	17	2,61	3,67	1,25	5	31	42	13	9	14	10	8	3,8	12
210	Female	24	4	39	22	1,94	3,67	2,5	4,4	32	41	9	10	11	10	13	3	9
211	Female	19	3	41	27	2,83	3	1,5	3,4	27	41	13	9	13	10	12	3,1	7
212	Female	17	1	64	7	1,06	1	1	1	15	25	7	11	11	14	12	3,7	12
213	Male	22	2	34	21	2	4,17	1,5	3,8	24	26	12	8	12	8	9	2,5	7
214	Female	18	2	51	14	2,39	2,5	2	3,8	18	35	6	10	13	13	11	2,9	9
215	Female	20	4	35	28	2,56	4,17	1,5	4,2	25	23	4	10	12	5	9	2,7	8
216	Female	23	3	43	26	2	5	1,5	2,4	30	36	11	9	11	7	9	2,7	9
217	Female	20	4	55	11	2,28	2,83	1,75	2,6	18	28	7	9	13	12	12	3,3	9
218	Female	17	1	50	15	2,28	2,5	1,25	2,2	25	38	8	10	11	8	11	3,2	9
219	Female	18	2	34	23	3,06	3,17	3,25	3,4	25	25	14	9	12	13	12	2,6	10
220	Female	19	2	38	24	2,56	3,17	2	5	23	24	8	7	9	5	11	2,6	9
221	Female	18	2	35	26	3	3,17	2	4,2	23	25	7	11	9	11	9	2,3	8
222	Female	19	2	30	30	3,72	3,67	2	4	26	24	5	8	7	2	6	1,8	7
223	Female	21	4	58	7	1,94	2	2	2	19	34	10	12	11	12	12	2,9	9
224	Male	19	2	53	12	3,22	3,67	3,75	3,4	29	38	14	7	9	8	14	2,6	8
225	Female	20	2	46	17	2,72	2,33	2	2,2	24	33	10	12	12	9	10	2,8	9
226	Female	17	1	49	20	2,56	2,67	1,75	2,4	23	32	10	11	10	7	11	3,1	8
227	Female	18	1	51	15	1,5	2,17	2	1	27	38	9	9	14	10	9	2,4	8
228	Male	44	1	56	7	1,94	1	1	1	22	33	9	8	13	12	9	3,5	9
229	Female	52	2	49	20	2,78	1	1	3,8	23	34	8	6	11	12	8	2,8	7
230	Male	46	4	44	19	2,78	1	2,75	4,6	21	32	9	6	10	9	8	3,1	7
231	Male	29	2	47	22	2,22	1	1	4	25	36	9	7	14	11	10	3	8
232	Male	30	1	57	8	1,94	1,5	1,5	1,2	17	27	3	5	11	12	7	2,9	11
233	Male	25	2	56	26	3	3,17	2,5	4	28	34	11	7	9	8	9	3,1	8
234	Male	44	4	55	12	1,94	1	1,5	1,2	14	19	6	7	14	13	10	4	7
235	Female	43	1	62	14	2,56	1,83	1,75	4	23	44	11	7	13	9	14	3,6	10
236	Male	35	1	54	16	2,28	2,5	1,75	2,4	20	37	12	8	9	13	10	3,2	9
237	Female	23	3	40	27	3,33	2,17	2,5	3,8	26	21	11	9	6	9	8	2,3	12
238	Female	18	2	30	34	3,44	1,83	4	4,8	30	31	5	6	12	4	12	2,4	8
239	Female	19	3	42	25	3,11	3,33	3	4,2	26	36	5	8	11	6	9	2,2	7
240	Male	23	1	63	9	2,06	2,67	1,75	3,2	18	33	13	9	13	14	13	3,6	12
241	Male	26	4	42	27	2,89	3	2	4,2	27	35	7	7	8	11	12	3	9
242	Female	31	4	49	22	1,89	3,83	1	3	27	43	6	8	14	9	9	2,6	9
243	Female	20	4	51	13	2,5	2,17	2	3,4	20	37	13	8	11	10	9	3,3	11
244	Female	19	4	57	16	2,56	3,67	2	4	20	34	9	7	12	11	10	3,5	9
245	Female	28	4	46	25	3,22	3,5	3	3,2	23	32	10	9	8	8	11	2,9	12
246	Female	20	4	46	30	3,28	3,33	2,75	3,6	23	27	4	8	13	8	6	2,6	8
247	Female	17	2	42	24	2,33	2,83	1,75	2,6	26	32	13	9	10	8	11	3,2	10
248	Female	21	4	46	26	3	2,67	2,5	2,8	23	34	2	9	13	11	8	2,7	6
249	Female	21	4	41	22	3,06	2,17	3	3,6	22	27	6	9	9	6	9	2,3	8
250	Male	25	1	55	12	2,17	3,17	3,75	3,4	19	41	13	11	12	13	11	3,6	9
251	Male	21	1	43	28	2,89	1,33	1	3,6	21	34	13	6	10	5	10	2,9	9
252	Female	21	1	48	16	1,72	2,67	2	2	17	28	5	10	9	13	11	3,5	10
253	Female	39	2	53	19	2,5	2,33	1,25	3,4	17	36	3	10	14	11	11	3	9
254	Male	17	1	55	18	2,56	1,67	1	2,4	24	40	8	7	13	13	8	2,9	7

255	Female	21	3	36	29	1,83	3,17	1,25	4	26	34	5	14	14	6	11	3,1	9
256	Male	19	1	47	21	2,72	3,33	2,25	3,4	26	28	11	7	11	10	8	3,1	6
257	Female	20	1	55	15	2,67	3,5	3	3,2	23	40	12	7	13	11	13	2,5	8
258	Male	21	1	43	25	3,06	2,83	2,75	2,2	31	29	6	5	6	12	10	2	9
259	Male	48	1	63	11	2	4,17	1,75	4,4	16	32	3	8	14	14	13	4	12
260	Female	16	1	52	20	2,78	2,17	2,25	4	31	34	9	7	10	3	11	2,4	9
261	Male	26	2	70	9	1,56	1	1	1,2	17	23	2	8	8	14	14	3,9	11
262	Male	17	1	37	19	3,61	2,5	3,25	4,2	33	27	7	8	5	8	3	1,6	6
263	Female	55	4	59	18	2,22	3	3	4	19	33	8	7	13	12	9	3,5	12
264	Female	50	2	56	15	1,94	1	1	1	16	26	11	8	14	14	12	3,9	12
265	Female	24	1	56	19	3	3,67	3,25	3,4	20	20	9	8	8	8	8	2,8	9
266	Female	17	1	52	15	2,78	3	1,5	1,2	19	37	7	6	9	6	11	3,2	9
267	Female	17	1	50	25	3,33	2,67	1,5	3,6	22	31	6	8	9	10	11	2,7	7
268	Female	18	1	51	19	2,89	3,67	2,75	3,6	25	35	13	8	12	7	8	2,7	12
269	Female	23	4	42	18	1,94	2,83	1,75	3	23	28	6	5	14	5	8	2,8	9
270	Female	22	3	54	13	2,39	2,17	1,75	2,8	21	45	9	8	14	8	13	3	12
271	Female	22	2	48	24	3,28	5	5	4,2	27	37	7	8	8	6	9	3	8
272	Female	20	3	54	14	1,94	3,17	2	2	20	37	13	8	14	13	14	3	12
273	Female	19	2	51	16	2,89	3	1,25	4	19	31	7	9	14	8	7	2,9	12
274	Female	19	1	53	17	2,39	4,17	1,5	3,4	23	37	8	9	14	9	12	3,3	8
275	Male	21	1	49	11	1,89	2,83	1,75	1	23	34	4	8	10	13	9	3,3	9
276	Female	17	1	52	27	3,28	3,17	3,5	4,2	24	35	11	8	12	5	11	2,6	6
277	Female	23	4	50	17	2,33	3,33	2,5	2,4	25	35	13	7	11	7	7	2,7	8
278	Female	25	4	45	32	2,72	4	3	5	29	43	9	9	9	7	9	2,8	12
279	Female	21	2	54	14	2,39	1,33	1,75	1,8	21	29	8	11	13	12	12	3,2	11
280	Female	22	2	46	27	3,94	4,5	5	4,2	26	36	7	8	8	7	9	2,8	6
281	Female	21	4	43	22	2,61	2	1,75	3,4	26	25	7	12	12	10	8	2,1	6
282	Female	21	3	50	26	3,39	4,67	1,75	5	26	37	13	13	9	9	14	2,6	8
283	Female	21	3	49	21	1,83	3	3,75	2,8	25	34	6	7	14	8	12	2,5	8
284	Female	19	2	47	26	3	4,17	3	4	22	34	5	6	12	8	12	3,1	6
285	Female	27	3	54	18	2,06	4	2	4	30	36	14	7	9	11	13	3	12
286	Female	18	1	48	18	2,56	2,33	1,75	2,8	22	39	10	9	14	7	9	3,2	12
287	Female	19	2	57	14	2,61	2,17	1,75	3	22	32	8	8	13	10	10	3,1	12
288	Female	20	3	45	21	2,78	1,83	2,5	1,6	29	40	6	10	13	5	8	2	12
289	Female	21	3	53	14	2,22	4,17	1,5	4	21	45	12	8	14	10	13	3,2	9
290	Female	21	1	44	26	3,17	2,83	3,5	4	23	33	3	6	9	6	6	2,3	9
291	Female	21	2	51	26	2,5	3,83	1,75	3,6	30	24	14	8	9	5	8	3	7
292	Female	21	3	51	23	2,06	2,83	2,75	4	22	36	14	7	13	8	13	3,6	12
293	Female	21	2	43	22	3,39	2,83	2,75	3	25	27	8	8	10	11	12	2,3	7
294	Female	18	1	38	22	2,89	3	2,75	3,6	24	31	8	8	12	6	8	2,3	8
295	Female	22	4	47	27	3,5	3,83	1	4	28	33	3	7	12	8	12	2,7	8
296	Female	19	2	33	34	3,33	3,67	1,75	4,4	27	27	2	8	11	3	14	2,4	6
297	Female	20	2	57	28	2,72	4,17	2	5	21	42	10	10	14	11	13	4	12
298	Female	18	2	55	14	1,56	3	1,25	3,6	32	44	6	8	10	11	13	3,2	8
299	Female	18	2	39	29	3,28	1,17	1	3,6	23	33	8	11	10	8	11	2,9	10
300	Female	17	1	51	19	3,22	3,17	2,75	3,6	25	38	12	9	13	5	9	2,6	12
301	Female	18	2	39	20	2,5	2	2	2,2	20	33	4	8	13	7	9	2,4	9
302	Female	18	2	41	25	2,83	4	1,5	4	21	22	9	10	8	8	10	3	6
303	Female	20	2	46	22	3,06	3	2,5	4	26	32	3	11	9	5	8	2,4	9
304	Female	17	1	57	20	2,17	3,33	1	2,6	16	27	10	9	11	13	13	3,8	11
305	Female	20	4	56	22	3,11	2	2,5	3,4	27	30	14	11	7	10	13	3,8	12
306	Female	20	4	52	15	1,44	3,83	2,25	2,8	27	33	12	6	12	8	9	3,2	11
307	Female	19	2	40	24	2,72	4	2,75	4	24	30	8	9	14	13	9	3	10
308	Female	21	2	47	23	3,22	4,5	3,25	5	31	40	7	7	14	6	10	2,4	11
309	Female	19	3	51	19	2,56	1	1,25	3	21	34	8	11	10	6	6	2,6	12
310	Female	23	4	46	28	3	3,33	3	3,2	28	27	5	11	9	7	10	2,5	9
311	Female	20	4	47	26	2,89	3,5	3,75	4,2	20	32	7	7	14	12	7	2,4	10
312	Neutral	18	2	52	14	2,39	2,33	1,75	2,6	23	35	6	9	13	9	10	3,2	8
313	Female	21	3	54	15	1,89	3,67	1	3,4	22	41	12	12	11	13	10	3,6	11
314	Female	19	2	49	24	3,06	2,67	1	2,2	24	35	7	9	12	6	11	2,5	12
315	Female	20	4	50	19	2,78	2,5	1,5	4,4	28	35	8	9	13	9	8	2,6	11
316	Female	20	2	37	26	2,61	1	1,5	3,2	29	36	4	11	12	7	9	2,7	10
317	Female	20	2	47	22	2,5	2,5	1	4	24	34	8	8	12	8	12	3,6	9
318	Female	19	1	28	30	3,78	3,17	2,75	5	30	22	2	9	9	3	7	1,2	6

319	Male	18	2	51	29	2,56	4,83	2,75	5	22	37	12	8	14	10	9	3,8	10
320	Male	27	4	33	33	3,5	5	2,75	4,6	23	25	8	5	7	5	11	3	8
321	Male	24	4	43	23	2,78	1,83	1	4,2	18	28	14	9	14	14	7	3,4	8
322	Male	24	5+	51	30	3,33	1,5	1,25	4,2	25	36	5	7	6	8	8	3,1	9
323	Male	20	2	57	26	2,83	4,33	1	4,8	18	38	7	8	10	11	10	3,6	10
324	Male	22	5+	38	24	2,5	1,17	1	2,4	28	21	12	7	9	12	13	3	11
325	Male	22	4	54	7	1,61	1,33	2,25	1	20	27	9	13	14	14	12	3,3	5
326	Male	22	4	42	21	1,83	3,17	1,75	3,4	18	36	13	7	10	13	7	3,2	11
327	Male	22	2	55	22	2,72	3,5	1,5	3,6	26	37	9	8	6	9	8	3,1	11
328	Male	19	1	50	13	2,44	2,17	2,5	2,8	18	23	7	7	12	13	9	3,2	11
329	Male	26	5+	58	20	2,17	3,33	2,75	5	27	35	11	5	9	11	10	3,1	10
330	Female	21	2	52	21	2,22	2,17	1	4,4	28	35	14	6	11	5	10	3,2	7
331	Female	26	4	45	22	2,72	3,33	1,5	4	23	26	3	8	9	8	10	2,5	8
332	Male	26	2	40	18	2,5	3,67	2,5	4	25	32	7	5	12	6	8	2,3	11
333	Male	26	2	52	18	2,78	4	1,5	3,4	23	26	9	7	10	11	12	2,6	10
334	Male	20	1	35	24	4,11	4,17	4,25	4,4	21	28	8	6	10	6	7	2,7	7
335	Male	19	1	48	21	2,56	1,67	1,75	2,4	19	30	12	8	10	11	13	2,9	12
336	Male	19	1	54	8	1,56	1,33	1	1	22	41	4	9	13	11	14	2,7	11
337	Male	18	1	53	10	1,28	3	1,5	3,6	24	34	7	8	12	8	10	3,1	8
338	Male	23	5+	47	21	2,89	3	1	4	28	31	11	5	10	12	10	3,6	12
339	Male	18	2	67	7	1,5	1	1	1	20	37	5	9	9	14	9	3,5	12
340	Male	22	2	52	18	2,61	3,17	2	1,6	22	37	4	8	9	14	8	2,7	9
341	Male	20	2	53	16	2,44	3	2,5	2	20	28	10	8	9	10	8	2,7	12
342	Female	31	2	52	28	3,94	4	4	4,8	27	30	8	13	9	8	8	2,5	6
343	Male	18	2	41	22	3,78	1,5	3,5	3	24	20	2	11	7	7	8	2,3	9
344	Male	21	2	54	14	2,11	1,83	1,75	1	20	34	5	6	11	14	6	3,6	11
345	Neutral	23	4	39	22	3,06	1,33	1,25	3,6	18	25	5	5	11	12	8	2,6	9
346	Male	25	2	58	13	2,56	2,33	1	2	22	33	14	5	8	10	11	3,4	11
347	Female	20	1	54	20	2,83	3,17	2	3	21	31	8	8	8	11	11	3	12
348	Male	20	1	60	14	2	2	1	2,6	27	40	9	8	12	11	12	3,3	12
349	Male	21	2	70	25	1,94	3,17	2,75	3	19	38	11	8	14	13	14	3,5	10
350	Male	23	4	62	5	2,56	1,67	1	2,6	15	20	9	8	14	14	12	4	12
351	Male	18	1	51	15	3,17	3	2,75	2,8	21	36	10	8	7	8	8	2,6	12
352	Male	20	1	56	12	2,28	1	1	1,2	18	21	8	8	11	10	9	3,1	9
353	Male	21	2	51	16	2,5	3	2	2,6	20	33	4	7	14	10	11	3,1	7
354	Male	18	1	69	5	2	2,67	2	2	12	24	14	9	10	12	12	4	12
355	Male	19	1	59	21	2,89	1,33	2,75	4	23	28	4	6	9	14	10	3,9	10
356	Female	17	1	55	16	2,78	2	1,25	3,4	18	33	12	7	10	12	12	3,3	12
357	Male	18	1	66	6	1,61	2,5	1,25	1,6	16	29	10	9	11	13	14	4	11
358	Male	18	2	46	24	2,72	2,5	1,75	3,6	23	41	10	7	9	11	12	3,3	7
359	Male	20	2	32	26	1,44	1,5	3	2,4	30	34	12	6	14	4	10	2,5	6
360	Male	19	1	55	22	4	3,67	3	4,6	29	33	12	9	8	9	11	2,9	8
361	Male	20	1	55	17	2,67	3,5	3,75	2,8	18	29	3	7	8	14	9	3,5	11
362	Male	19	1	52	20	2,67	3,5	3,5	3,6	36	32	7	7	8	5	8	3,3	10
363	Male	21	1	56	15	2,39	1,67	1,5	1,4	22	38	5	8	11	12	9	3,1	9
364	Male	22	1	54	22	3,28	2,33	3	3,4	33	43	10	7	10	5	10	2,6	10
365	Male	20	1	48	19	2,67	4,17	2	1,2	25	33	13	9	11	9	8	2,5	12
366	Male	25	4	56	14	1,89	2	3	3	17	22	4	7	11	12	14	2,8	9
367	Male	21	4	14	26	4,83	4,5	5	4,4	33	26	5	3	8	2	10	1,8	3
368	Male	19	1	49	14	2,5	1	3,25	2,4	23	30	6	9	14	8	13	3,2	7
369	Male	20	1	52	11	2,83	3	1,75	3,4	19	30	8	5	11	10	10	3,4	9
370	Male	17	1	52	21	3,44	1,33	1,75	1,4	37	40	7	8	8	8	8	2,3	10
371	Male	17	1	59	15	2,17	1	1	1	22	22	10	8	8	12	10	3,2	12
372	Male	17	1	36	20	3	3	3	3	19	31	9	6	8	8	9	2,9	10
373	Male	18	1	52	17	3	3	3	3	27	36	8	8	8	8	8	2,8	9
374	Male	20	1	50	11	2,5	1,67	2,25	2,6	17	21	4	6	11	11	10	3,2	10
375	Male	18	1	56	6	1,94	2,67	1,75	2	21	29	7	5	11	12	8	3,3	10
376	Male	18	1	53	13	2,61	3,17	3	3	20	28	7	10	6	13	10	3,5	9
377	Male	18	1	65	15	2,83	3,5	4,5	2,8	32	30	6	10	7	6	10	2,3	7
378	Male	19	2	61	13	1,89	1	1,75	3	20	35	12	6	14	14	11	3,9	10
379	Male	17	1	63	3	1,94	1	1	1	15	29	8	8	9	14	10	3,8	12
380	Male	22	2	39	16	2,39	3,5	2,75	3,2	28	33	7	8	8	11	10	2,3	8
381	Male	19	1	36	28	3,61	3,83	3,5	4	31	26	8	9	7	5	11	2,2	7
382	Male	22	1	56	13	3	3	3	3	21	27	8	8	8	8	8	3,5	12

383	Female	23	3	54	20	2	2,83	1	2,6	23	36	9	9	7	11	8	2,9	12
384	Female	20	3	51	22	2,89	2,17	3,5	3,2	32	41	8	7	8	6	12	2,9	9
385	Female	19	1	60	15	2,67	3,17	1,25	4	19	42	7	12	12	11	11	3,6	11
386	Female	22	3	38	29	3,28	4,17	2	4,2	30	27	9	9	7	6	13	3,4	8
387	Male	27	3	49	24	2,22	3,5	2	3,4	22	39	8	7	11	8	9	3,3	9
388	Female	21	4	32	29	3,11	4,5	4,5	5	24	34	8	14	14	9	12	3,5	8
389	Male	25	3	48	19	2,78	3,33	2,5	2,8	23	42	4	8	9	5	13	2,7	9
390	Male	17	1	44	24	2,56	1,5	1,5	2	21	29	8	9	9	4	10	3,1	8
391	Female	21	3	42	18	3	2,83	3,5	4,2	27	39	5	8	6	4	11	2,4	7
392	Female	20	2	42	21	1,94	3,17	2,25	2	25	29	9	12	6	11	14	3	8
393	Female	19	3	58	10	2,61	3,83	1,5	4,4	20	43	8	8	9	10	14	3,2	12
394	Female	18	1	46	12	2,5	4,17	3	3,8	21	33	2	13	12	13	12	2,5	5
395	Female	20	3	55	12	1,44	2,67	1,25	3	16	41	11	11	11	7	10	3,4	12
396	Female	18	2	55	11	1,67	1,33	1	3	18	36	3	8	14	13	7	3,8	10
397	Male	21	2	55	9	2,5	3	1,5	3,8	19	32	4	9	11	14	12	3,6	10
398	Female	21	4	51	20	2,94	3	3,25	4	23	35	10	6	13	5	7	2,7	9
399	Female	21	3	58	16	2,39	2,67	2,25	3,6	19	38	9	9	14	13	11	3	12
400	Male	20	2	49	17	2,22	2,83	1	3,4	25	27	11	8	12	9	12	3,3	12
401	Female	19	1	43	22	2,28	2,17	1,75	2	23	26	6	7	9	4	8	2,6	6
402	Male	25	2	40	26	3,78	4,83	2,75	4,8	32	33	10	9	9	7	9	3,2	7
403	Female	20	2	54	17	2,56	3,5	2,5	3,2	25	36	9	11	11	12	10	3,5	12
404	Female	26	2	53	12	2,33	3,67	1,5	2,6	21	37	11	8	13	11	12	3,4	12
405	Female	19	2	46	22	2,83	2,83	3	3	26	27	9	8	8	8	8	2,3	6
406	Male	21	2	48	16	2,67	2,33	2,25	1,8	20	26	10	8	13	13	11	3,4	11
407	Male	21	3	41	26	1,67	3,83	1	3	24	31	2	7	14	11	13	2,6	6
408	Female	24	4	47	26	2,28	4	2,5	4	27	35	11	8	10	12	12	3	9
409	Male	21	1	39	26	4	3	4	4,6	25	35	4	9	14	8	9	2,5	8
410	Female	23	3	35	34	3,67	4,33	4,25	3	32	30	2	11	6	2	8	1,8	7
411	Female	21	2	53	14	2,94	3,33	3,75	3,4	25	31	11	8	12	9	12	3	11
412	Female	22	2	50	16	3	2	1	2,2	17	28	11	9	9	11	10	2,9	12
413	Male	20	2	57	12	2,78	4	2	3,6	26	46	13	9	14	8	10	3,3	10
414	Female	21	3	41	26	3,5	2	3	4,8	31	28	5	4	9	2	13	2,5	9
415	Female	18	1	42	22	2,33	3	3,25	2,8	18	20	5	10	12	9	13	3,1	9
416	Female	20	2	56	21	2,72	4,83	2,25	4,2	26	39	10	9	10	12	14	2,8	12
417	Female	19	2	42	26	2,83	3	1	4	31	31	6	7	13	5	13	2,3	9
418	Male	23	2	42	17	2	2,17	2,5	1,4	30	39	3	9	8	7	11	3	11
419	Female	20	2	34	27	2,56	4,33	5	3	35	34	4	10	10	6	12	1,4	7
420	Male	21	3	70	18	1,11	2	2,25	2	13	13	14	8	14	14	14	4	9
421	Male	20	2	60	9	2,33	3,67	3	3,4	20	32	9	13	11	13	11	3	9
422	Female	22	4	54	15	2,33	3,83	3	2,8	26	37	14	8	14	12	12	3,4	12
423	Female	21	2	57	13	2,61	4	2,75	3,2	24	34	10	8	9	10	8	2,9	10
424	Female	20	2	49	25	2,56	4,33	2,25	4	25	41	9	9	14	7	11	3,1	12
425	Male	23	2	55	10	2,06	3,5	2,5	4	23	39	8	7	12	14	10	3,1	8
426	Female	20	2	53	21	2,06	4,5	2,75	2,4	26	38	13	9	14	8	11	3,3	11
427	Female	18	2	32	32	3,67	3,83	3,75	5	37	39	13	8	13	3	11	2	11
428	Female	20	2	43	30	3,33	3,5	3	3,8	27	32	12	8	12	8	12	2,9	9
429	Female	18	2	58	11	2,17	2,83	1	2,6	21	26	8	8	6	12	14	3,1	8
430	Female	21	2	44	32	2,94	4	1,75	4,4	27	19	9	8	9	3	10	2	8
431	Female	18	2	51	19	2	2,5	3,25	2,4	21	42	13	9	13	12	13	3,4	12
432	Female	20	2	47	22	1,28	4,67	3,5	4,2	22	32	7	3	12	7	13	2,9	11
433	Female	20	2	48	37	3,78	3,67	2,5	4,2	40	28	4	6	9	3	11	1,7	5
434	Female	21	1	42	23	3	4,17	3	4,8	25	41	8	7	11	5	13	2,8	8
435	Male	19	1	46	21	2,56	3,5	2,5	2,6	30	29	10	8	9	10	14	3,1	10
436	Female	19	2	57	19	2,11	1,83	3,25	2,4	29	41	14	12	14	9	14	3,7	12
437	Female	20	2	29	33	3,61	4,33	4,5	5	32	29	2	6	11	12	14	2,3	3
438	Female	22	4	36	35	3,56	2,33	3,75	4,4	31	32	7	9	9	7	10	2,5	11
439	Female	18	2	47	19	3	2,33	1,75	4	17	31	12	8	14	7	12	3	9
440	Male	20	2	59	10	2,06	2,33	1	1	22	30	11	9	13	14	14	3,9	12
441	Female	19	2	55	18	2,33	3	2,25	3,2	41	41	10	8	14	9	12	3,1	10
442	Male	19	2	48	19	2,72	3,17	2,75	2,6	13	13	6	6	8	11	9	2,9	9
443	Male	23	2	45	21	3,72	3,67	2,75	4,6	28	35	12	8	7	10	11	2,8	6
444	Male	18	2	48	26	2,67	3,67	1	2	21	32	4	9	9	12	7	2,9	11
445	Male	18	2	50	19	3,06	1	1,75	3,4	25	27	8	10	9	8	10	2,9	12
446	Male	19	2	38	25	3,11	2,5	3	3,8	30	32	2	4	7	12	11	2,7	8

447	Male	19	2	53	17	2,72	3,33	2,25	2,2	25	32	6	10	10	12	9	3,3	9
448	Female	20	4	52	12	3,22	1,83	1	3	19	44	5	10	14	13	10	3,3	12
449	Female	17	1	41	26	3,78	3,67	3,25	4,6	26	32	4	8	12	6	9	2,2	8
450	Male	21	4	50	25	3,5	4	3	3,8	25	34	4	9	10	13	11	3	9
451	Female	19	2	45	19	2,33	1,83	1,25	3,2	18	22	7	10	10	9	7	3	10
452	Female	20	2	54	15	2,28	2,33	1,5	1,4	21	37	8	7	11	6	13	3,1	9
453	Male	20	2	49	17	3,06	4,17	2,75	4,4	29	34	8	12	7	7	10	2,4	9
454	Male	20	1	50	14	2,17	2,5	2,75	2,2	29	35	9	10	10	8	7	3,1	10
455	Female	20	3	56	10	2,17	3,33	2,75	2,4	19	37	7	9	14	10	9	3,6	12
456	Female	19	2	57	18	2,78	3,83	2,5	3,4	25	39	12	9	10	8	13	3,4	11
457	Female	20	2	53	23	3,11	3,83	2,75	3,2	25	46	9	11	6	8	14	3	12
458	Female	19	2	42	23	2,61	2,33	3	3	36	36	3	9	8	7	9	3	7
459	Male	22	2	40	22	3,61	3,17	4,25	2,6	26	30	12	6	11	9	14	2,2	11
460	Male	23	3	45	24	3	5	5	5	36	32	3	12	6	10	9	2,8	8
461	Female	19	1	59	14	2,22	1,83	1	3,2	20	36	9	11	14	8	14	3	11
462	Female	20	2	31	29	3,72	1	3,25	4,4	23	34	14	10	9	6	10	2,3	11
463	Female	18	1	63	12	1,89	3,33	1,75	4,2	23	38	9	11	12	13	12	3,2	10
464	Male	19	1	50	13	2,67	2,5	2,75	2,8	21	27	6	5	8	8	6	3,1	10
465	Male	19	1	41	29	3,28	4,5	3,5	4,2	30	40	13	9	13	6	12	3,2	8
466	Female	25	1	41	29	3,78	4	4	4,4	35	31	9	7	8	3	12	2,4	4
467	Female	21	2	34	26	3,78	3,67	2,25	4,2	30	32	13	12	10	10	8	2,5	10
468	Female	18	1	34	27	3,5	2,17	1,25	4,6	29	27	8	6	9	7	9	2,4	11
469	Female	22	2	38	28	2,94	4,17	2,25	3,6	26	26	2	10	8	6	7	2,3	7
470	Female	21	1	54	16	1,89	3,83	1,25	3,4	24	42	11	9	13	8	10	3,1	12
471	Male	24	2	38	26	3,39	3,33	2	4,6	29	38	8	2	9	11	13	2,6	9
472	Male	23	1	55	16	2,28	2,83	1	3,2	16	21	9	8	11	14	9	3,6	10
473	Male	22	4	54	18	2,67	2,5	1,5	3,8	26	28	11	5	13	9	10	3	9
474	Male	21	2	55	18	2,78	2,67	2	2,6	26	33	8	7	12	12	8	2,9	9
475	Male	19	1	49	14	2,44	2,83	1,5	2,6	18	27	9	14	8	9	13	3,2	10
476	Male	19	1	54	12	2	2,33	2,5	2,2	19	33	9	11	8	12	12	2,9	10
477	Male	20	2	53	22	2,78	3,33	3,25	3,6	22	37	11	8	12	11	12	3,4	10
478	Female	20	2	46	16	2,67	2,17	3,5	3,4	29	40	4	8	12	8	10	2,4	10
479	Female	18	1	51	20	2,44	4,17	2	2,8	18	35	10	10	12	11	14	2,6	6
480	Female	23	1	47	22	3,39	2	1,5	3,4	24	32	6	10	9	7	10	2,4	7
481	Female	19	1	45	32	3,61	4,83	5	3,4	33	30	3	8	9	5	11	1,6	9
482	Male	20	2	44	22	2,28	4	3,25	4,6	31	34	9	8	12	12	11	3	8
483	Female	20	2	57	11	1,61	3,17	1	3,8	19	48	11	9	8	12	14	3,4	12
484	Female	18	2	43	26	1,94	2,83	1	2,4	21	30	9	12	12	9	11	3,6	12
485	Female	19	1	59	14	3,44	2,5	3	2,8	30	34	6	8	12	7	4	2,2	8
486	Female	19	1	38	22	2,06	4,17	4,25	2,6	30	26	13	5	8	6	12	2,4	8
487	Female	17	1	50	15	2,61	1,5	1	3,2	25	32	9	9	9	9	11	2,6	11
488	Female	18	2	42	17	2,5	2,83	1	2,4	13	25	7	9	12	9	9	2,8	9
489	Male	18	2	58	22	2,94	2,5	1,5	2,4	28	41	8	4	8	10	13	2,9	12
490	Male	22	2	53	13	1,83	3,5	2	3,8	26	44	7	7	11	13	12	3,1	10
491	Female	22	3	54	14	2,94	2,67	2,5	2,8	26	33	12	9	11	11	14	3,1	12
492	Male	20	2	34	26	3,78	3,5	4,5	4	24	20	3	13	7	12	7	2,1	6
493	Male	18	2	51	15	3,5	1	2,25	4,6	26	30	5	7	8	6	12	2,3	9
494	Male	21	2	50	15	2,28	1,33	1,75	3,6	25	36	9	8	13	12	10	2,8	9
495	Male	18	1	61	8	1,83	3,67	1,5	1	24	36	9	12	14	14	11	3,8	11
496	Female	18	1	48	16	2,56	2,5	1,25	1,2	21	31	8	8	11	8	7	2,6	9
497	Female	19	2	24	37	3,5	4,83	3,25	4,6	32	30	4	10	12	8	7	2,2	6
498	Female	18	2	49	22	3,06	2,83	3,5	4	26	33	8	8	8	5	13	3,3	9
499	Female	26	2	38	22	2,33	1,67	1,75	3,2	22	37	9	8	10	7	14	3,2	8
500	Male	21	5+	50	22	2,89	3,83	2	4,2	24	31	13	6	14	11	9	3,3	11
501	Male	19	2	40	26	3,44	2,33	1,25	4	20	31	7	11	11	12	11	2,8	6
502	Male	21	4	44	23	2,89	2,5	2,5	4,4	20	39	14	8	8	9	13	2,9	9
503	Male	23	1	51	15	1,72	2,17	1	1	31	47	9	8	6	8	8	2,3	11
504	Male	17	1	39	24	3,11	1,83	2,25	1,6	27	31	11	7	4	14	14	3,3	12
505	Male	20	3	57	13	1,5	2,5	2,75	3,8	28	38	7	7	10	14	7	3,4	12
506	Male	17	1	30	31	2,94	3,5	3,75	2,6	29	22	8	9	8	3	6	2,3	3
507	Male	22	4	53	16	2,28	3,5	3	3,8	21	29	9	9	12	13	11	3,2	10
508	Male	21	4	55	24	2,94	1,5	1,5	3,8	22	40	6	8	7	13	8	2,8	7
509	Male	19	1	51	14	2,5	3,33	2	2,6	18	25	9	6	10	9	11	3,4	10
510	Male	24	4	48	21	2,67	3,67	3,25	2,8	26	28	11	9	10	7	9	2,4	9

511	Male	21	2	39	25	2,89	3,33	1,25	3	27	27	4	9	13	13	9	2,4	7
512	Male	25	4	43	24	1,72	1,67	3,25	2	27	34	10	5	7	6	12	2,3	6
513	Male	20	1	56	18	2,94	2,33	2,75	3	22	27	7	9	12	9	9	3	8
514	Female	21	4	37	27	3,61	3,5	3,25	3,2	29	21	10	7	8	7	11	1,9	11
515	Male	22	3	53	10	2,72	2,83	1,25	1,8	24	32	4	8	7	14	5	2,7	12
516	Male	18	1	55	25	2,28	2,5	1	2,8	23	36	9	10	12	11	12	3,2	12
517	Male	20	4	44	27	3,5	2,67	2,5	4	35	24	3	8	2	8	13	2,1	6
518	Male	23	4	54	13	2,5	2,5	2,25	3	17	33	9	8	10	12	12	3,3	10
519	Male	20	1	56	9	2,39	2,33	2	2,6	24	28	9	9	11	8	11	2,9	11
520	Male	23	2	43	22	3	2,33	1	3,4	23	29	7	8	9	8	7	2,2	9
521	Male	17	1	44	18	3,06	2,17	1,5	3,2	24	27	8	8	5	6	9	2,7	8
522	Male	21	4	48	20	2,83	2	2,25	3	25	30	3	8	11	8	12	2,3	9
523	Male	21	4	50	13	2,5	1,5	1	2,4	25	25	8	8	9	13	13	3,4	12
524	Male	18	1	59	11	2,28	2,67	1,5	1,2	23	38	12	7	14	10	6	3,7	12
525	Female	21	4	39	13	3,44	4,17	2,5	4,6	28	37	8	9	12	10	6	3,3	8
526	Male	23	4	53	10	2,56	1,33	2	1,8	19	29	7	6	10	12	9	3,2	8
527	Male	18	2	40	23	2,72	2,17	2,25	3	25	28	5	8	8	9	11	2,7	7
528	Male	21	3	42	25	2,89	3,83	2,5	4,2	29	27	8	9	12	11	11	2,7	9
529	Male	19	3	58	12	2,22	3,5	2	2	16	26	5	7	14	14	9	3,5	9
530	Male	21	5+	52	27	3	2,33	1	5	28	44	14	10	14	11	11	4	10
531	Male	17	1	48	13	2,94	2,5	1,5	3,8	22	28	13	7	8	12	9	3,1	8
532	Male	21	1	52	14	2	2,17	2,25	1,4	26	45	7	8	8	13	13	3,7	12
533	Male	19	2	53	16	2,72	2,33	2	4	23	34	8	8	11	12	12	2,9	8
534	Male	22	2	53	11	2,28	3	1,5	2,2	18	33	9	12	10	13	11	3,2	12
535	Male	19	2	54	24	3,11	4	3,25	4	21	24	10	7	12	12	9	2,5	9
536	Male	20	1	59	14	2,44	3,67	1,5	3,6	20	33	11	7	12	12	13	3,7	12
537	Male	18	1	52	18	2,78	2	2,5	2,4	20	31	5	6	9	12	12	3,2	10
538	Male	17	1	55	18	3,33	1,67	2	4,2	25	24	5	9	13	11	10	2,3	9
539	Female	17	1	55	14	2,33	2,83	2	3,2	27	31	6	5	12	11	11	2,9	12
540	Male	21	4	49	16	2,44	3,17	2,5	1,8	22	37	13	6	8	11	12	3,4	11
541	Male	17	1	45	21	2,33	1,17	2,5	3,4	27	23	8	5	4	10	7	2,9	6
542	Male	23	2	29	27	3,5	2,33	1,75	4,4	25	21	2	11	8	3	8	1,2	4
543	Female	22	4	48	23	2,72	2,17	1,5	4	24	37	10	10	12	9	13	2,8	12
544	Female	23	1	54	21	2,5	2,5	1	3,4	19	31	11	9	13	12	13	2,9	12
545	Female	41	2	47	25	2,44	2	2	2	29	48	9	9	11	12	13	3,7	12
546	Female	22	4	46	24	1,83	3,83	2,75	3,8	25	39	10	7	13	8	13	3,5	12
547	Female	36	4	68	14	2,28	1	1,75	3,8	19	41	14	8	14	14	14	4	12
548	Male	23	3	38	30	3,44	3,67	3,75	3,6	36	35	8	8	8	7	8	2,1	7
549	Female	22	3	32	31	3,78	3,83	3,75	5	26	32	10	9	14	10	11	2,3	8
550	Female	24	3	43	21	3,17	4,17	2,25	4,2	21	31	7	9	14	7	10	2,5	6
551	Female	22	4	68	16	2,11	3,5	1	2,8	22	45	8	10	13	6	13	3,1	10
552	Female	20	4	44	20	3,33	2,83	3,25	2,6	36	37	13	4	9	7	8	3	12
553	Female	23	4	49	25	3,72	4	2,5	4,4	26	34	8	10	8	10	9	2,7	9
554	Female	21	3	42	21	2,17	3,67	3,75	4	22	34	11	9	9	13	9	3,5	12
555	Female	19	2	51	22	2,83	3	2	3,8	23	36	12	11	10	10	13	3,3	8
556	Female	19	3	61	17	2,67	3,17	2,5	2,6	20	44	14	8	13	9	11	2,7	11
557	Female	19	3	33	33	3,17	1	1	1	29	29	4	8	7	2	12	1,3	6
558	Female	18	1	28	26	3,83	3,67	4,25	5	31	27	2	6	13	2	7	1,3	4
559	Female	40	3	55	10	2,22	1,33	1,75	3,8	21	38	14	8	14	14	9	3,8	12
560	Male	18	2	50	14	3,28	4	3,75	4	23	28	9	8	10	12	10	2,5	8
561	Female	22	3	47	26	2,89	1,83	1,75	3,2	29	35	13	9	7	5	14	2,7	12
562	Female	20	3	43	25	2,06	2,67	1	2	27	38	5	7	13	8	9	2,4	12
563	Female	22	2	46	26	2,61	4	2	2,8	21	29	5	7	14	6	10	2,3	6
564	Female	18	1	52	14	2,61	2,83	2	3,2	24	29	13	5	14	9	11	3,3	9
565	Female	20	3	45	23	3,22	3,33	1,5	5	26	37	9	9	14	13	11	2,6	11
566	Female	20	3	46	24	3	1,67	1,5	2	29	36	10	8	9	6	11	2,6	12
567	Male	25	4	56	15	2,39	3,67	2,5	2,4	21	38	12	7	13	12	11	3,5	8
568	Female	21	2	52	12	1,94	4,17	1,5	1	22	38	11	7	14	11	9	2,9	12
569	Female	30	4	47	23	2,78	3,17	2	3,4	24	28	7	8	14	3	7	2,4	6
570	Female	19	3	45	26	2,78	3,67	1,5	4	26	35	13	9	13	8	13	3	12
571	Female	23	4	53	20	3,33	2,67	2,25	3,6	21	31	9	9	10	9	10	3,1	10
572	Female	18	2	40	25	2,94	2,5	2,75	3,4	25	34	9	8	12	6	4	2,4	11
573	Female	47	3	51	18	2,28	2,17	1,5	3,8	22	26	8	7	10	13	7	2,9	7
574	Female	43	1	55	17	2,56	2,17	2,5	3,6	21	37	14	7	14	7	10	2,4	9

575	Female	19	2	41	24	2,5	3,5	2,25	2,2	31	29	11	10	12	7	10	2,6	8
576	Female	21	3	26	35	4,17	2,17	5	5	32	30	3	8	14	6	8	1,9	9
577	Female	20	2	54	23	3,33	3,33	3,75	4,4	24	35	14	10	13	10	11	3	12
578	Female	21	3	37	29	3,5	4	2,75	5	31	33	13	7	10	3	8	2,1	8
579	Female	19	3	55	11	2,39	1	1	1,6	16	25	9	9	13	12	9	3,6	11
580	Male	25	3	29	31	3,17	3,67	3,5	4,6	29	20	10	5	8	7	8	2,5	9
581	Female	20	3	52	14	2,33	2,33	1,75	2,6	17	31	5	9	12	11	10	2,8	9
582	Female	45	2	69	7	2,17	1,33	1	2,4	17	31	12	9	13	14	9	4	11
583	Male	18	2	43	19	3	3,5	3	3,8	25	33	12	11	10	11	11	2,7	9
584	Female	20	2	48	15	2,72	1,67	1,25	1,2	20	36	5	12	14	13	7	2,4	11
585	Female	26	3	45	26	3,28	4,5	2,75	3,4	26	27	13	9	4	9	7	2,4	7
586	Female	19	2	45	24	2,44	2,17	2	3,6	20	35	11	9	14	5	10	2,5	8
587	Female	22	3	60	29	2,33	3,33	2	4,4	30	40	11	9	6	8	13	2,9	9
588	Male	22	4	49	25	2,5	1,83	2	3,8	22	36	7	10	11	14	6	3	12
589	Female	21	4	58	17	3,06	2,33	2	3,2	19	38	4	9	12	9	6	3,1	12
590	Female	20	2	57	27	2,39	3,17	1	4	25	44	9	9	9	8	13	2,6	7
591	Female	21	3	41	23	2,56	1	2,25	4	26	30	6	11	10	8	8	2,1	11
592	Female	18	2	40	22	3,11	2	1,5	3	27	27	9	8	11	5	8	2,2	8
593	Female	23	4	59	17	2,06	4,33	2	1	24	36	14	9	14	7	12	3,1	10
594	Male	52	4	49	14	2,33	1,83	2	2,2	18	34	9	8	5	8	8	3,1	10
595	Female	21	1	54	12	2,33	1,5	1,25	2,4	28	39	14	6	12	11	10	3,3	11
596	Female	21	1	55	11	1,94	1,5	1,5	1,6	21	37	12	6	13	10	10	3,5	9
597	Female	30	2	56	10	1,5	1,33	1	1	20	39	10	7	13	10	8	3,3	12
598	Female	38	3	54	23	2,94	1	2,5	4,2	23	43	4	6	12	7	8	3,3	12
599	Female	20	1	56	23	2,61	2,17	2	2,8	29	36	14	9	14	10	13	3	12
600	Female	36	3	60	10	2,72	1,33	1,75	4	20	37	11	9	14	13	9	3,8	11
601	Female	20	2	59	9	2,17	1,67	1	1,8	19	31	5	8	14	12	9	3	12
602	Female	23	3	52	16	2,5	3,67	1,5	3,4	23	39	8	8	10	10	13	2,7	11
603	Female	22	4	53	15	2,89	4,17	2	3,2	21	37	13	8	9	12	11	3,3	12
604	Female	23	4	45	28	3,78	4	2,5	5	25	33	10	8	10	7	10	2,8	9
605	Female	21	3	51	29	3,28	1,33	1,5	4,2	31	34	10	8	11	5	10	2,5	10
606	Female	24	4	34	27	3,28	2,17	1,75	3,6	21	26	3	7	9	6	9	2,7	6
607	Male	26	4	66	16	2,5	3,17	3	3	19	29	6	9	10	13	8	3,3	12
608	Female	37	3	60	20	3,06	1	2	3,8	17	37	4	12	14	13	10	4	8
609	Female	21	2	49	16	2,61	2	1,75	2,6	19	33	12	7	9	12	13	2,8	9
610	Female	24	4	55	26	2,33	1	1	3,4	19	19	11	9	13	11	12	2,8	12
611	Female	21	2	44	22	3,11	2	2,25	2,8	19	27	9	8	10	9	9	2,5	9
612	Female	51	3	64	14	2,33	2,5	2,25	2,6	19	40	10	6	9	11	10	3,1	8
613	Female	21	2	48	25	3,33	1,83	3,75	3,8	32	35	14	9	10	9	8	2,7	12
614	Female	23	2	44	22	3,22	3,5	1,75	4	21	23	8	8	13	10	12	2,8	9
615	Female	20	2	53	25	2,61	3,33	2,5	3,8	24	32	13	8	14	9	14	2,6	7
616	Female	21	1	57	29	2,89	3,17	3,5	4,2	31	35	8	7	10	7	12	2,6	9
617	Female	22	5+	62	10	1,94	1,33	1	1,4	17	39	14	8	14	14	9	3,9	11
618	Female	21	3	57	14	2,61	3	2,25	3	19	32	10	7	10	12	11	2,8	11
619	Male	23	3	31	28	3,33	4,17	3,25	4,8	25	24	3	10	11	6	6	1,5	7
620	Female	21	5+	38	26	3,72	4,67	1	5	31	33	7	8	11	3	7	2,3	8
621	Female	22	3	47	29	3,17	2	1	3,6	29	40	8	9	13	10	12	2,8	12
622	Female	20	2	50	12	2,17	3,5	1	2,4	20	36	14	8	10	13	12	3,3	12
623	Female	21	4	48	15	2,56	1	1	1	20	34	9	8	12	7	10	3,2	12
624	Female	18	2	55	16	2,5	3,17	1,5	3,6	20	47	13	9	9	13	14	3,3	9
625	Female	21	2	52	15	2,11	2,17	2,5	1,8	23	39	11	7	14	6	6	3,5	9
626	Female	18	2	49	29	3,33	3,83	3,75	3,6	25	26	11	7	10	7	10	2,4	8
627	Female	19	2	54	18	2,78	4,33	3,75	3,2	25	33	10	6	11	7	11	2,8	9
628	Female	19	3	45	28	2,33	3,5	1,75	3,4	24	29	10	8	13	8	14	2,9	11
629	Female	21	3	54	18	3,11	1,67	2	3	24	30	9	10	12	8	8	3	9
630	Female	19	3	53	18	2,61	1,67	1	1	22	45	8	10	10	14	12	3	12
631	Female	21	3	34	33	3,39	3,83	1,75	5	26	33	9	8	11	12	12	2,4	7
632	Female	19	3	52	22	3,22	3,17	2,5	4	24	27	10	8	11	10	10	2,9	9
633	Female	19	3	41	20	3,06	1	1	3,8	26	27	8	10	7	7	8	2,3	8
634	Female	19	3	51	21	2,56	2,83	1,5	3	21	30	4	6	13	8	9	2,7	7
635	Female	19	3	35	25	3,39	2,17	1	3,8	25	33	5	12	11	8	9	2,8	7
636	Female	23	3	35	27	4	3,17	2,75	4	24	28	6	10	8	5	9	2,8	10
637	Male	22	3	49	13	2,5	2,67	1,5	3,6	26	33	8	12	9	10	10	3	11
638	Female	21	3	50	22	2,72	3	3,25	3,4	20	28	10	8	10	10	11	2,9	9

639	Female	22	4	42	25	2,83	3	2	4	26	36	8	11	8	3	9	2,8	6
640	Female	20	3	48	25	2,56	3,83	2,75	4	31	37	11	8	12	10	9	3,1	12
641	Female	20	3	32	22	3,28	3,17	3	4	27	24	3	12	8	3	9	2,4	6
642	Female	21	2	53	27	2,11	3,67	2,25	3	30	39	11	6	13	4	9	3	8
643	Female	19	2	47	14	2,72	2,5	1,5	3,2	19	26	10	8	10	10	8	2,3	8
644	Female	18	2	43	22	2,94	3,5	3	3,8	25	33	7	8	11	8	11	2,7	8
645	Female	18	2	35	28	3,67	4,33	3	4	22	31	6	11	8	2	7	1,9	9
646	Female	21	2	38	30	3,28	2,83	3	2,8	28	24	7	9	8	7	13	3,1	8
647	Female	55	1	56	13	2,33	3	3	2	22	36	10	8	11	12	9	3,5	11
648	Female	23	3	33	30	3,94	3,5	4,25	5	29	24	8	9	5	10	7	2,5	9
649	Female	23	3	40	24	3,11	4	2,75	4	24	32	10	8	12	5	12	2,1	8
650	Female	19	1	60	6	2,17	2	1,75	2,8	20	42	13	9	9	13	13	3,8	12
651	Female	20	4	52	19	2,72	1,33	1,75	2,8	27	37	7	9	10	8	11	2,7	10
652	Female	25	4	50	26	2,78	4,17	4,25	5	23	34	2	9	12	9	8	2,9	10
653	Female	21	4	55	22	3,22	1,83	3,75	4	20	27	7	9	12	10	11	2,8	7
654	Female	19	4	48	23	2,67	3	2,25	4	26	37	8	8	11	8	7	3,2	12
655	Female	18	2	53	17	1,78	1,5	1	2	21	35	9	9	14	13	13	3,7	11
656	Female	23	2	43	23	2,78	3	3	3	20	25	9	8	12	10	8	2,9	9
657	Female	18	2	52	35	2,72	2,83	2,75	4,4	28	35	9	6	12	3	7	3,1	10
658	Female	23	1	37	29	3,61	4	2,5	4,2	28	43	10	10	10	3	7	2,9	7
659	Female	18	2	43	28	3,22	3	3,25	3,6	18	32	10	9	13	6	12	2,1	8
660	Female	20	2	53	13	2,56	2,5	2,25	2,2	18	27	9	9	8	11	13	3,1	9
661	Female	21	2	33	29	2,94	3,5	1	3,2	23	24	8	12	8	4	9	1,9	7
662	Female	18	2	39	23	2,83	2,67	2,25	2,2	23	30	7	8	10	9	7	2,8	9
663	Female	18	2	53	9	1,89	3	3,75	3,8	22	33	5	8	12	11	9	2,6	9
664	Female	19	2	46	23	2,78	3	3,25	2,4	26	32	9	8	10	7	11	2,2	8
665	Female	18	2	45	20	3,28	3,67	4,25	3,4	27	32	7	10	11	5	7	2,8	8
666	Female	19	3	44	23	3	3,33	2	4	19	29	13	8	9	8	10	2,8	10
667	Female	19	3	52	27	3,11	3,5	2,5	4	20	38	10	10	13	12	11	2,9	12
668	Female	20	3	67	17	2,33	1	1,5	3	18	32	9	9	14	13	12	3,8	12
669	Female	23	3	42	22	2,89	4	2,5	4	20	27	13	8	14	8	9	2,7	12
670	Female	21	3	28	36	3,89	4,17	3,25	5	33	23	8	6	10	6	10	1,8	6
671	Female	21	3	26	38	3,78	4,5	3,25	5	29	21	3	13	6	2	7	1,6	6
672	Female	19	3	54	16	2,72	3,33	2,25	3,6	19	36	10	9	9	9	10	3	9
673	Female	18	2	56	15	2,28	3,17	2,75	3	22	31	12	12	13	13	12	3	11
674	Female	22	1	52	14	2,28	1,17	1,75	2,2	24	32	9	8	12	11	7	3,4	9
675	Female	19	3	47	26	3,11	3,17	2,75	4	25	34	6	7	11	7	8	2,8	12
676	Female	19	1	27	33	2,89	4,33	3,5	4	22	24	8	10	10	3	10	2,1	7
677	Female	22	5+	49	25	3	4,17	2,25	3,2	27	28	8	8	13	9	10	3	9
678	Female	22	4	50	27	2,61	2,67	1,25	4,8	18	36	10	8	14	12	12	3,3	10
679	Female	17	1	42	27	2,94	2,5	1	3,4	27	28	10	9	13	8	12	2,4	12
680	Female	20	2	53	11	2,28	3,67	1	3,6	22	39	8	12	13	10	10	3,1	9
681	Female	18	2	55	18	2,5	2	1,25	3,2	19	39	8	9	12	9	8	2,9	12
682	Female	33	2	56	14	2,89	2,83	2	2,8	23	40	7	7	11	8	10	2,5	12
683	Male	19	2	53	18	3	2,33	2,25	2,4	19	26	8	9	9	10	9	3	12
684	Female	19	2	55	13	2,56	2,33	1	2,2	21	38	13	8	10	10	8	3,6	9
685	Female	19	2	45	23	2,61	3,5	2,75	3,4	20	25	4	10	9	10	6	2,5	9
686	Female	19	2	38	19	2,67	3,17	4,75	2,2	22	27	8	6	12	9	9	2,3	8
687	Female	19	3	58	17	2,17	1,83	1	2,2	22	38	13	8	14	11	11	2,9	12
688	Female	17	1	57	16	2,44	3	1,5	3,8	19	32	9	7	13	11	10	3,7	9
689	Female	16	1	39	24	3,06	3,17	3	2,6	21	34	10	8	7	9	11	2,7	12
690	Female	21	4	62	21	3,11	3,5	2,25	3,2	21	44	13	8	14	7	13	3,6	10
691	Female	21	4	41	19	3,17	2,83	2,5	4,2	26	37	5	6	10	10	9	2,6	9
692	Female	21	2	34	26	3,89	3,67	2,75	4,6	28	27	11	8	8	10	8	2,3	8
693	Female	17	1	51	14	2,78	3,83	2,25	3,6	23	32	5	7	9	11	5	2,9	9
694	Female	19	3	46	23	3,56	2,5	3,25	3,8	25	34	9	8	11	11	8	2,9	9
695	Female	18	2	47	28	3,33	3	4	3,8	19	26	9	6	14	10	7	2,7	8
696	Female	21	2	43	18	2,78	3,5	3	3,4	27	38	7	9	13	13	10	2	9
697	Female	20	2	62	19	2,11	3,5	1,75	3,4	21	48	12	8	13	7	13	3,9	11
698	Female	22	4	52	16	2,44	3	1	3,2	20	34	10	9	14	11	12	3	12
699	Female	23	4	50	17	2,33	3,17	1	1,6	23	32	12	7	7	10	9	2,6	9
700	Female	22	4	44	29	2,72	3,17	2,5	3,8	23	26	5	6	11	4	10	2,3	7
701	Female	20	2	56	8	1,89	1,83	1	1,2	18	31	12	10	12	13	12	3,2	12
702	Male	22	4	53	16	2,72	3,5	4	1,6	23	32	7	8	14	6	12	2,7	8

703	Female	20	4	46	26	2,33	3,5	2,75	3,6	26	34	4	8	12	12	11	3,2	10
704	Male	21	3	38	19	2,89	2,67	3,5	3,6	21	24	5	8	13	7	7	2,7	3
705	Female	17	1	55	13	2,06	1,83	2	2,2	26	31	11	10	12	11	14	3,3	11
706	Male	18	1	42	20	3,11	2,67	3,25	3,8	18	22	2	6	14	13	8	2,6	7
707	Male	17	1	60	7	2,33	1,5	1,25	2,4	23	31	5	9	12	11	8	3,6	9
708	Male	17	1	54	5	1,61	2,83	1,5	3	21	32	4	7	11	12	14	3,4	10
709	Female	21	2	43	27	2,83	3,5	2,5	2,6	26	19	2	8	9	7	12	2,2	9
710	Male	18	1	57	9	2,17	1	1	1,6	25	39	10	9	14	14	12	3,5	12
711	Male	18	1	51	18	1,94	2,17	2,25	2,2	27	27	7	5	7	5	12	2,7	8
712	Male	18	2	61	12	2,22	1,5	1,5	2	22	32	9	6	12	10	13	3,1	8
713	Male	20	2	65	5	1,67	2,33	1,5	1	20	27	7	8	9	14	10	3,9	12
714	Male	19	3	39	26	2,89	1,83	1,75	3,6	22	31	4	9	14	8	9	2,6	9
715	Female	21	1	32	24	2,39	5	3,5	4,2	25	33	11	6	9	7	12	2	12
716	Male	17	1	59	17	2,5	1	1,25	2,8	29	34	8	4	8	10	3	3	8
717	Male	18	2	41	13	2,11	1,17	2,75	3	18	26	4	6	11	13	10	2,8	8
718	Male	21	2	52	11	3,17	4,33	2,25	1,6	20	34	2	9	9	12	11	2,5	8
719	Female	19	3	48	20	3,06	2,5	2	3,6	17	33	10	10	12	14	13	3,1	12
720	Male	22	2	32	28	2,78	2,67	3	4,2	21	27	13	7	8	8	13	2,9	7
721	Female	22	4	32	35	3,78	4,5	4,25	4,6	32	28	13	7	9	7	11	2,1	9
722	Female	23	1	47	32	2,39	5	3,5	3	19	37	8	8	10	7	10	4	12
723	Male	19	1	49	20	2,67	3,83	2	2,8	31	39	11	7	10	9	13	3,1	11
724	Male	21	3	51	14	2,28	4,67	3,5	3	16	24	4	8	7	10	14	3,1	9
725	Male	19	3	52	21	2,56	3,67	2,5	2,8	25	27	12	7	11	10	9	2,6	6
726	Female	19	3	30	33	4	3,33	2,5	5	18	28	3	8	12	2	13	1,5	3
727	Female	21	3	38	21	2,61	2	2,5	2,4	31	42	8	12	8	8	8	2,1	10
728	Female	18	1	53	25	2,28	3,5	3,5	3,6	20	18	9	8	12	11	8	2,6	9
729	Female	19	2	49	19	2,06	4,17	4,25	2,8	31	40	14	7	9	4	10	2,1	12
730	Female	21	2	38	29	2,56	3,5	2	3,8	27	25	7	10	11	6	12	2,7	4
731	Female	19	2	53	18	2,61	3,33	1,75	4	21	30	7	9	14	9	12	3,3	10
732	Female	21	3	43	20	2,61	2,33	1	3,6	22	36	9	11	13	8	12	2,7	11
733	Female	22	2	22	24	4,22	5	3,5	4,8	46	29	5	8	6	2	14	1,8	12
734	Female	28	4	55	25	3,28	3,33	1,75	3,6	30	45	14	9	9	9	12	3,5	12
735	Male	25	4	41	31	2,72	1,5	2	3,8	32	37	13	7	8	6	13	3	12
736	Female	17	1	56	8	2,28	3,5	2	3,2	21	36	11	8	12	11	9	3,1	12
737	Female	20	2	50	14	3	2,67	2,75	2,8	25	32	10	9	12	9	7	2,7	8
738	Female	20	1	42	27	3,67	4,33	3	3,8	23	23	8	8	8	6	7	2,4	6
739	Male	19	1	50	22	2,5	3,83	2	2,2	26	30	9	7	11	4	11	3,2	7
740	Male	24	4	44	18	2,67	4,17	3,25	2,4	18	33	7	7	11	9	10	3	6
741	Female	20	1	55	13	2,44	4,17	2,5	3	20	33	7	8	9	11	9	3,2	11
742	Female	24	4	32	20	3,06	3,83	2,5	3,2	23	32	4	8	11	7	8	2,5	7
743	Female	18	1	50	24	2,5	4,5	3	3,2	28	37	4	8	12	7	8	2,3	9
744	Male	21	3	50	23	3,22	3,67	3,25	4	17	35	8	8	10	9	11	2,5	9
745	Female	19	2	45	25	3	1	2	2,8	26	29	3	7	13	10	13	2	6
746	Female	19	2	41	21	3,39	3,67	4	3	28	28	7	10	10	6	8	2,4	9
747	Female	20	2	51	25	2,33	3,17	2,5	2,2	23	33	9	9	10	10	11	2,9	9
748	Female	21	1	44	26	2,83	4,5	3,5	4,4	25	33	11	8	13	5	14	3,4	9
749	Neutral	19	1	48	28	2,5	3	2,25	2,6	36	43	8	8	8	10	14	2,2	9
750	Male	26	4	36	35	3,11	3,5	3,5	3,4	32	34	14	8	11	6	14	2,5	7
751	Female	21	1	53	22	2,78	4,5	3	3,4	22	37	10	10	13	4	14	3,8	8
752	Female	20	2	43	25	3,17	3,33	2,75	2,6	33	29	5	10	9	5	11	1,7	11
753	Female	20	2	60	13	2,11	3	1,5	2,4	25	41	13	10	14	9	12	3,1	12
754	Female	23	1	36	34	2,56	3,67	2,25	3,2	27	34	11	4	5	4	10	1,7	9
755	Female	24	2	40	23	2,78	3,67	3,25	3,6	26	26	13	12	11	6	12	2,8	10
756	Male	23	3	42	19	1,67	3	1,75	3,2	23	36	9	8	14	12	13	2,9	11
757	Female	20	2	35	27	2,56	3,67	3	2,4	25	26	3	8	9	5	11	2,6	8
758	Female	21	3	32	28	3,39	5	5	5	21	22	8	8	10	8	9	2,7	10
759	Female	22	4	52	19	3	3,33	3	3,8	22	33	7	8	12	7	11	3,2	12
760	Female	20	1	30	36	3,11	4	1,75	4	31	30	6	14	11	5	12	1,5	8
761	Female	18	2	36	32	2,67	4,17	4,5	3,8	34	30	8	8	13	2	13	1,6	6
762	Female	18	2	43	31	2,83	2,67	3,25	3,4	20	33	9	9	12	8	12	2,7	9
763	Female	18	2	36	26	3,5	3	2,75	4,8	34	20	11	6	11	6	13	2	7
764	Male	19	2	36	28	3,17	3,83	2,75	3,6	22	29	3	10	9	7	10	2,7	8
765	Female	21	2	40	18	2,5	2,83	1,5	3	24	27	12	7	6	10	10	2,4	5
766	Female	19	3	42	18	2,06	2,67	1,25	1,4	23	37	5	7	14	7	13	3	12

767	Male	22	1	43	19	2,11	3,5	1,25	2,8	22	29	8	8	13	4	9	2,4	7
768	Female	20	4	51	21	2,44	2	1,5	3,6	30	39	8	7	11	5	8	2,8	10
769	Female	18	1	69	3	2,56	2,83	2,25	3	19	41	10	9	13	14	13	4	12
770	Female	19	1	52	19	3	4,17	2,75	2,8	24	39	7	12	9	6	10	2,2	11
771	Female	20	2	52	31	3,39	2,67	2,75	5	36	33	7	8	14	7	11	2,5	10
772	Female	24	2	48	13	2	3,5	1,5	3,2	20	34	12	8	14	8	13	3,3	12
773	Male	18	1	55	10	2,28	3	1,5	2,6	18	33	14	9	10	13	7	3,3	10
774	Male	21	4	65	7	1,5	1,67	1	1	22	41	8	7	14	14	10	3,9	12
775	Female	21	3	51	8	2,44	3,17	1	4,6	23	41	2	9	10	3	9	3,1	11
776	Female	19	1	59	10	1,5	1,5	2,5	3	19	44	12	8	14	8	14	3,8	10
777	Male	24	2	57	14	3,06	1,5	1,5	3,4	19	33	10	7	11	13	11	3,6	9
778	Female	20	2	56	16	1,72	2,67	1	3	14	27	9	7	13	9	11	3,6	12
779	Female	20	2	45	18	2,11	2,33	2,5	2	19	30	8	8	12	10	10	2,9	7
780	Female	20	2	52	18	2,33	3,5	4,5	3,6	23	31	10	6	13	6	9	2,6	7
781	Female	19	3	52	29	2,83	2	1	1,8	27	29	12	10	7	10	12	2,9	9
782	Female	18	2	40	21	2,22	4,17	2	4,8	24	35	12	10	12	7	10	3,2	7
783	Female	19	2	37	31	3,67	4	4	5	21	30	10	9	9	8	8	3	9
784	Male	28	3	41	26	2,78	4,17	2,75	4,8	25	35	7	5	13	13	14	2,8	11
785	Female	24	4	56	15	2,39	2,5	3	3,4	21	30	11	9	9	11	10	3	10
786	Female	18	1	50	16	2,83	1,33	1	4,2	25	33	7	9	13	8	8	2,7	11
787	Female	22	3	50	19	2,94	3	3,25	3,8	26	33	9	8	13	8	6	2,9	9
788	Female	20	3	44	24	2,28	4,33	2,5	3,4	28	32	7	8	12	5	9	3,2	7
789	Female	19	1	62	10	2,22	3,5	1	1,2	21	40	7	10	13	12	11	3,4	12
790	Female	19	1	31	29	3,22	4,33	3,25	5	28	22	9	8	11	4	9	2	9
791	Female	19	1	48	27	2,39	2,33	1,5	2,8	19	29	7	7	12	7	10	2,9	12
792	Female	19	1	55	19	3	2	1	3	28	38	9	8	10	10	12	2,9	12
793	Female	19	1	48	15	2,44	2,83	1	1,8	23	40	11	7	13	11	12	3,6	9
794	Male	18	2	51	16	1,83	3,67	1,5	3	20	30	13	8	5	6	12	3,2	9
795	Female	19	1	47	26	2,61	1	1,75	2,2	24	32	13	9	10	3	14	2,2	12
796	Female	21	2	50	23	2,22	2,17	1,25	4	22	33	7	9	13	10	11	2,7	12
797	Female	19	1	43	19	2,89	3,67	1,5	3,8	21	30	10	9	12	8	12	2,7	8
798	Male	19	1	56	10	1,67	1,33	1	2,8	17	29	14	9	12	13	12	3,6	12
799	Male	25	4	46	21	2,11	1	1,75	2	24	36	5	9	13	9	10	3	9
800	Female	27	2	52	21	3	3	2,75	3,4	25	39	6	8	10	6	10	2,3	8
801	Male	27	1	42	29	2,33	3,83	1,75	4	27	29	12	9	8	12	13	3	12
802	Female	19	1	47	15	2,33	2,67	2,25	2,6	27	30	10	8	8	5	12	2,5	9
803	Female	19	1	56	12	2,28	2,5	1	1	22	38	13	10	10	9	11	3,3	12
804	Female	23	4	48	23	2,83	2,33	1	3,6	18	32	4	6	14	7	11	3,5	6
805	Male	20	1	59	4	2,39	1,83	2,5	2,2	18	22	11	8	10	12	10	3,2	8
806	Female	20	2	33	30	3,67	3,5	3,25	5	24	27	8	8	10	8	10	2,4	10
807	Male	20	1	54	11	2,44	2	1,25	2	16	26	13	7	13	9	13	3,6	12
808	Female	21	2	39	26	3,11	4,5	2,5	2,2	25	32	8	9	10	6	9	2,3	9
809	Female	21	2	41	22	3	1,67	2,25	2,2	29	31	11	9	13	11	12	3,2	12
810	Female	20	1	53	11	2,28	2,67	1,25	2,8	18	31	13	8	14	11	10	3,1	12
811	Female	20	1	54	23	3,17	3,83	4,25	3,8	24	31	6	7	6	12	13	2,4	6
812	Female	22	4	54	20	2,33	1,33	2,75	3	17	29	10	9	9	8	12	3	12
813	Female	23	4	43	18	2,44	1	1,75	1,4	21	37	7	7	14	3	14	3	9
814	Female	22	3	55	20	2,78	3	1,75	2,8	16	33	11	8	9	9	12	3,3	11
815	Female	20	2	53	17	2,06	2	2	2,8	19	31	11	9	12	10	10	3,1	9
816	Male	20	1	53	16	2,17	2,83	1	4	17	23	8	8	11	11	8	3,1	8
817	Female	20	2	56	16	1,44	2,5	1	1	20	34	6	8	13	11	9	3,4	9
818	Female	20	2	29	22	2,39	1	1,75	1,6	20	28	10	10	12	8	12	3	12
819	Male	33	2	58	18	2,94	3,67	3,75	3,8	29	34	12	6	12	12	12	3,3	12
820	Female	24	4	57	12	1,94	1,83	1	2,8	18	42	6	8	14	12	13	3,5	12
821	Female	18	1	48	19	2,89	3,67	1,75	4	30	40	7	9	14	11	11	2,8	8
822	Female	17	1	53	16	2,39	2	2,25	4	20	37	13	8	14	8	14	3	12
823	Female	18	2	43	23	2,44	2,17	2,5	4	26	33	9	7	10	6	8	2,7	7
824	Female	17	1	49	16	2,33	1,83	1,5	2,6	20	39	8	8	14	9	11	3,2	8
825	Male	19	2	49	10	1,17	1	1	2	24	40	14	8	14	10	13	3,8	12
826	Female	21	2	44	27	3,33	4	3,25	4,8	24	29	14	8	4	11	8	2,4	7
827	Male	21	2	34	32	2,67	1	1,75	3,2	30	31	8	4	10	5	10	2,7	6
828	Female	19	2	47	14	1,94	1,67	1,5	2,2	24	28	9	8	12	12	10	3,1	12
829	Female	18	2	43	11	1,83	1	1	3,4	23	35	9	8	13	13	13	3,3	9
830	Female	18	2	49	22	2,56	1,67	2	4,4	21	42	14	7	9	11	8	3,3	10

831	Male	18	2	43	23	2,5	3,83	1,75	4,4	22	27	10	9	10	7	10	2,7	8
832	Male	17	1	44	24	2,83	3,5	2,75	4,6	24	26	6	7	10	10	10	2,6	8
833	Female	17	1	60	15	1,94	3	1	4	18	23	8	8	13	7	9	3,1	10
834	Female	17	1	58	17	2,61	3,33	1,75	4,6	23	31	13	9	13	14	12	3,4	12
835	Female	19	2	45	23	2,33	2,5	1,5	4,2	27	41	12	9	14	9	13	3,4	7
836	Female	18	2	48	22	2	1,33	1,5	2,4	23	29	7	9	14	9	12	2,7	10
837	Female	19	2	42	27	3,28	2,5	1,5	3,6	25	27	9	9	9	8	12	2,2	9
838	Female	20	2	62	35	3,33	4,83	3,25	4,8	32	30	9	9	14	9	12	2,1	4
839	Female	23	4	51	25	2,94	2,17	1,5	3,8	23	35	10	8	12	7	11	2,8	12
840	Female	17	1	54	16	2,22	3,5	1,25	3,8	23	38	13	9	11	10	10	3,2	12
841	Female	19	3	54	11	2,11	3,17	1,75	3,8	22	37	10	8	13	10	13	3,8	11
842	Female	19	2	35	28	3,89	2	3	3,4	28	30	8	10	9	2	6	1,7	8
843	Female	18	1	52	20	2,5	1,67	1	1,6	30	31	8	9	5	3	6	2,4	11
844	Female	20	4	59	16	2,67	3,67	1	3,6	25	38	12	7	13	8	12	3,4	11
845	Female	21	3	37	30	3,39	3,33	3	3,8	26	30	9	10	9	7	10	1,9	8
846	Female	19	1	55	12	2,11	2	2	2	22	32	11	9	14	13	11	3,1	12
847	Female	18	1	43	20	2,89	1,83	1	3,2	20	29	9	8	11	13	8	3	9
848	Female	19	2	49	18	2,28	3,83	3	3,6	23	33	13	9	13	13	10	3,2	10
849	Female	18	2	50	28	1,89	4,83	1,75	3	25	29	8	6	8	9	13	3	5
850	Female	19	2	55	22	2,39	1,5	1	2,8	25	42	14	8	14	12	14	4	11
851	Male	18	2	60	12	2,22	1,33	1,75	2,8	21	33	9	7	12	11	10	3,2	9
852	Female	21	4	53	11	2,06	2	1	3,6	19	40	8	7	13	8	6	3,9	10
853	Female	18	1	58	22	2,94	1,33	1	4	25	38	11	8	9	7	12	2,8	12
854	Female	18	1	59	9	2,11	2,17	1,5	2	17	34	8	7	13	12	8	3,2	10
855	Female	17	1	61	12	2,5	1,67	1,5	1	21	43	11	10	13	12	13	3	12
856	Female	22	2	44	30	2,89	3,67	3,5	3,6	26	33	12	9	11	10	10	2,7	9
857	Female	19	1	58	13	2,39	1	1	2,6	19	24	10	8	14	12	9	3,3	9
858	Female	26	2	57	19	2,78	2,33	1,25	4,2	28	39	12	8	11	7	12	2,9	12
859	Female	41	2	65	14	1,44	2,67	2	3	23	46	11	10	13	10	10	3,8	12
860	Female	22	2	52	20	3,06	1,5	2,5	3,2	26	41	10	9	9	5	4	2,7	5
861	Female	21	1	22	31	3,89	1,83	4	5	31	22	3	9	13	7	9	1,3	3
862	Female	41	2	55	9	2,22	1,83	1,25	2	17	30	12	12	9	11	6	3,1	12
863	Female	21	2	39	21	3	3	3	3	30	27	9	8	8	8	8	2,6	6
864	Female	34	2	43	22	3	3,17	2,25	3,6	37	30	12	7	9	7	10	2,5	9
865	Female	31	1	55	17	2,22	2,67	2,25	3,4	24	34	13	7	14	12	12	3,3	11
866	Female	35	2	47	24	2,39	3,5	3,75	3,8	25	34	10	7	11	8	11	3,2	7
867	Female	22	2	53	14	3,33	1,67	2,75	3,6	17	37	6	7	13	7	13	2,7	10
868	Female	24	2	51	20	2,11	1,33	1	1,6	27	31	12	9	12	8	12	2,9	11
869	Female	47	2	57	14	2,67	1,83	1,5	3	21	41	12	8	13	14	12	3,7	10
870	Female	20	2	48	15	2,5	3,17	2,25	3,4	20	40	13	7	12	14	7	3,4	11
871	Female	40	2	55	11	2,17	1,67	1,5	1,2	16	27	13	9	11	13	13	3,5	9
872	Female	21	1	36	32	3,94	1,67	3,75	4,6	25	16	6	9	14	9	11	1,6	5
873	Female	18	1	53	18	2,56	3,33	2	4	19	26	7	9	8	13	9	3,1	9
874	Female	29	1	39	19	2,72	3,5	4,25	3,8	31	24	7	9	11	6	11	2,4	9
875	Female	30	2	59	18	2,11	2,5	1,75	3	23	46	11	7	12	12	13	2,7	12
